# Role of Virally Encoded Circular RNAs in the Pathogenicity of Human Oncogenic Viruses

**DOI:** 10.3389/fmicb.2021.657036

**Published:** 2021-04-20

**Authors:** Janardhan Avilala, David Becnel, Ramsy Abdelghani, Asuka Nanbo, Jacob Kahn, Li Li, Zhen Lin

**Affiliations:** ^1^Tulane University Health Sciences Center and Tulane Cancer Center, New Orleans, LA, United States; ^2^Department of Medicine, Tulane University Health Sciences Center, New Orleans, LA, United States; ^3^National Research Center for the Control and Prevention of Infectious Diseases, Nagasaki University, Nagasaki, Japan; ^4^Institute of Translational Research, Ochsner Clinic Foundation, New Orleans, LA, United States

**Keywords:** circular RNA, circRNA, oncogenic virus, EBV, KSHV, HPV, MCV, HBV

## Abstract

Human oncogenic viruses are a group of important pathogens that etiologically contribute to at least 12% of total cancer cases in the world. As an emerging class of non-linear regulatory RNA molecules, circular RNAs (circRNAs) have gained increasing attention as a crucial player in the regulation of signaling pathways involved in viral infection and oncogenesis. With the assistance of current circRNA enrichment and detection technologies, numerous novel virally-encoded circRNAs (vcircRNAs) have been identified in the human oncogenic viruses, initiating an exciting new era of vcircRNA research. In this review, we discuss the current understanding of the roles of vcircRNAs in the respective viral infection cycles and in virus-associated pathogenesis.

## Introduction

Circular RNAs (circRNAs) are a class of non-linear macromolecules that are single-stranded covalently closed cyclized RNAs with a great diversity in length, varied from less than 100 to thousands of nucleotides (Kolakofsky, 1976; Sanger et al., 1976; Hsu and Coca-Prados, 1979; Arnberg et al., 1980; Salzman et al., 2012, 2013; Jeck et al., 2013; Memczak et al., 2013; Jeck and Sharpless, 2014; Rybak-Wolf et al., 2015; Kristensen et al., 2018). The first evidence of circRNA was reported in 1976 when Sanger et al. conducted the electron microscopy analysis of the denatured RNA molecules from the viroid of the pale fruit disease of cucumber (CPFV). The authors found that these viroid RNA existed as single-stranded circles (Sanger et al., 1976). Three years later, endogenous circRNAs were further detected in the cytoplasmic RNA extracted from human HeLa cells, monkey CV-1 cells, and Chinese hamster ovary cells as well as total RNA isolated from *Physarum polycephalum* (a plasmodial slime mold) (Hsu and Coca-Prados, 1979). However, at that time, the synthesis of circRNAs was considered to be a rare event and those circRNAs were treated as “splicing noise” and byproducts likely resulting from splicing errors (Cocquerelle et al., 1993). Hence, the potential impact of this discovery did not receive broad recognition and only a handful of follow-up studies were conducted in the next three decades.

The dawn of the circRNA era occurred approximately 30 years later when high-throughput next-generation sequencing technology and associated informatics approaches have been utilized in the circRNA research field. Since the early 2010s, thousands of endogenous circRNAs have been identified in all domains of life including human, animals, plants, yeasts, bacteria, and viruses (Danan et al., 2012; Salzman et al., 2012, 2013; Jeck et al., 2013; Memczak et al., 2013; Jeck and Sharpless, 2014; Rybak-Wolf et al., 2015; Kristensen et al., 2018; Tagawa et al., 2018; Toptan et al., 2018; Ungerleider et al., 2018, 2019a; Zhao et al., 2019). To date, the list of identified circRNAs is still rapidly growing and circRNAs are now appreciated as an important evolutionarily conserved component of living organisms.

Circular RNAs are usually expressed in a tissue- and/or developmental stage-specific manner (Memczak et al., 2013). The mature circRNAs can be detected in cytoplasm, nucleus, as well as extracellular vesicles (e.g., exosomes) (Li Y. et al., 2015). In general, circRNAs show ∼100–1,000 times lower abundance than their associated isogenic linear RNAs and are generated by more than 20% of expressed genes (Huang et al., 2017). However, in many cases, circRNAs can be the main gene products and exhibit more than 10-fold higher expression levels compared to related linear RNAs (Jeck et al., 2013). Lacking free termini, circRNAs are resistant to the hydrolysis by numerous cellular exonucleases such as RNase R, and thus have significantly longer half-lives compared to their linear RNA counterparts (Jeck et al., 2013).

**FIGURE 1:**
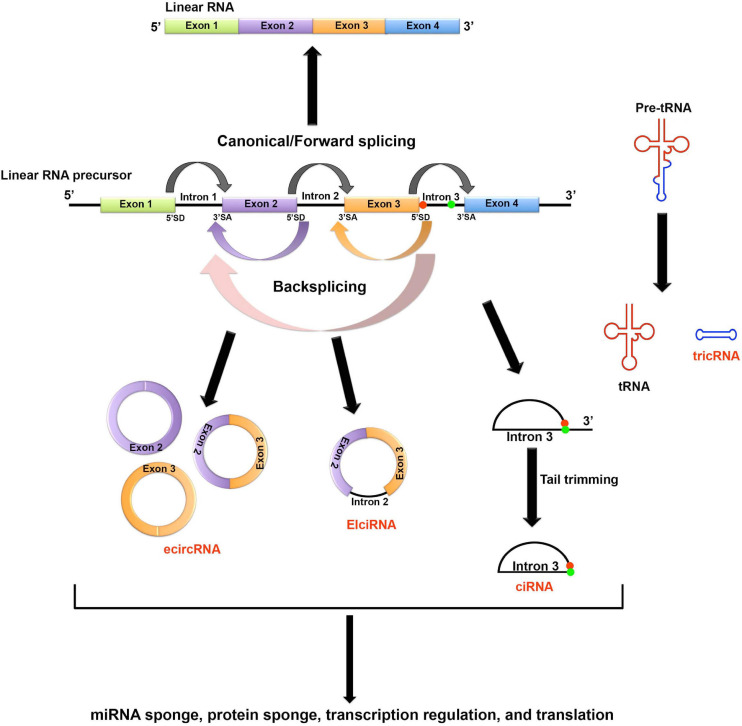
Biogenesis and potential functions of circRNAs. Based on the parental gene component that’s carried by the circRNAs, circRNAs can be classified to four major types. (1) The most common type is the exonic circRNAs (ecircRNAs) which contain only one or multiple exons; (2) The exon-intron circRNAs (EIciRNAs) carry both exons and introns; (3) The circular intronic RNAs (ciRNAs) carry introns only; and (4) The tRNA intronic circRNAs (tricRNAs) are formed by circularization of the excised tRNA introns. The formation of ecircRNAs and EIciRNAs requires a special type of splicing known as backsplicing, which allows a downstream 5′ splice donor (SD) to react with an upstream 3′ splice acceptor (SA). Thus, a 3′–5′ phosphodiester bond can be established to circularize the RNA molecule. ciRNAs are generated by lariat introns removed from pre-mRNAs by canonical splicing. A consensus 7-nucleotide GU-rich sequence near the 5′ splice donor (shown as red dot in the figure) and an 11-nucleotide C-rich sequence near the branchpoint site (shown as green dot in the figure) can help prevent the lariat from debranching and exonuclease attacking. Stable ciRNAs can then be formed after trimming the 3′ tail downstream from the branchpoint. Lastly, tricRNAs are formed by directly linking the free ends of introns excised from the pre-tRNAs. circRNAs can function as molecular sponges to sequester miRNA and protein. In general, circRNAs mainly function as non-coding regulatory transcripts. However, some circRNAs can be translated through either IRES-mediated or m^6^A-mediated non-canonical cap-independent translation initiation.

Based on the parental gene component that’s carried by the circRNAs, circRNAs can be classified to four major types (Figure 1)(Jeck et al., 2013; Zhang et al., 2013; Li Z. et al., 2015; Noto et al., 2017): (1) the most common type is the exonic circRNAs (ecircRNAs) which contain only one or multiple exons; (2) the exon-intron circRNAs (EIciRNAs) carry both exons and introns; (3) the circular intronic RNAs (ciRNAs) carry introns only; and (4) the tRNA intronic circRNAs (tricRNAs) are formed by circularization of the excised tRNA introns. The detailed mechanisms of generating different types of circRNAs are still less understood. The current theory holds that the formation of ecircRNAs and EIciRNAs requires a special type of splicing known as backsplicing, which allows a downstream 5′ splice donor to react with an upstream 3′ splice acceptor. Thus, a 3′–5′ phosphodiester bond can be established to circularize the RNA molecule (Jeck et al., 2013; Li Z. et al., 2015). Unlike exon-derived circRNAs, ciRNAs are generated by lariat introns removed from pre-mRNAs by canonical splicing. A consensus 7-nucleotide GU-rich sequence near the 5′ splice donor and an 11-nucleotide C-rich sequence near the branchpoint site can help prevent the lariat from debranching and exonuclease attacking. Stable ciRNAs can then be formed after trimming the 3′ tail downstream from the branchpoint (Zhang et al., 2013). Lastly, tricRNAs are formed by directly linking the free ends of introns excised from the pre-tRNAs (Noto et al., 2017).

In general, the exon-derived circRNAs (ecircRNAs and EIciRNAs) are produced from pre-mRNA transcribed by RNA polymerase II. The circularization signals are located within the introns adjacent to the circularizable exons (Jeck et al., 2013; Zhang et al., 2014). A growing number of RNA-binding proteins (RBPs) as well as spliceosome components are reported to be involved in the circularization process, including adenosine deaminases acting on RNA (ADAR), the splicing factor muscleblind (MBL), quaking (QKI), DExH-Box helicase 9 (DHX9), epithelial splicing regulatory protein 1 (ESRP1), FUS, serine/arginine-rich proteins, nuclear factors NF90/NF110, small ribonucleoprotein particle U1 subunit 70K (snRNP-U1-70K), small ribonucleoprotein particle U1 subunit C (snRNP-U1-C), pre-mRNA processing 8 (Prp8), cell division cycle 40 (CDC40) (Chen and Yang, 2015; Conn et al., 2015; Ivanov et al., 2015; Kramer et al., 2015; Rybak-Wolf et al., 2015; Aktas et al., 2017; Errichelli et al., 2017; Yu et al., 2017).

In recent years, the way in which circRNAs execute their function has been the subject of active study. Although the biological significance of the majority of the identified circRNAs are still unclear, it’s believed that circRNAs predominantly function as regulatory non-coding RNAs (ncRNAs) (Guo et al., 2014). circRNAs can function *in trans* or *in cis*. For instance, there is increasing evidence showing that some circRNAs (such as CDR1as/ciRS-7) can serve as microRNA (miRNA) sponges to sequester endogenous miRNAs and modulate miRNA function *in vivo* (Hansen et al., 2013; Piwecka et al., 2017; Kleaveland et al., 2018). Meanwhile, Ashwal-Fluss et al. (2014) have shown that some circRNAs can be co-transcribed with their associated linear mRNAs in a competitive manner. Thus, these circRNAs can function as an RNA trap for mRNA synthesis by competing with linear splicing *in cis*. Since circRNAs can bind to numerous RBPs, it’s postulated that these stable RNA molecules can serve as decoys, scaffolds, or transporters for many RBPs and regulate their functions (Du et al., 2016; Holdt et al., 2016; Abdelmohsen et al., 2017; Pamudurti et al., 2018). The discovery of circRNAs in the extracellular compartments such as exosomes indicates that circRNAs might also function as secretory intercellular signaling molecules to affect distant cells (Li Y. et al., 2015). Interestingly, several recent studies have shown that certain circRNAs (e.g., circZNF609) can be translated in a non-canonical cap-independent manner (Legnini et al., 2017; Pamudurti et al., 2017; Yang et al., 2017). Although the biological impact of circRNA translation and the resulting products are still largely unclear, it’s postulated that the truncated protein products/peptides may compete with the full-length protein products encoded by their associated linear mRNAs and affect relevant signaling pathways (Legnini et al., 2017). Together, this evidence suggests that circRNAs may function as important gene regulators and modulate signaling transduction at different levels.

Cancer is developed through a multi-step and multi-factorial process. As an emerging gene regulator, circRNAs are expected to be involved in the regulation of various carcinogenic pathways. To date, there is emerging evidence showing that disruption of circRNA expression correlates with a broad spectrum of human cancers, including hepatocellular carcinoma (HCC; Qin et al., 2016; Shang et al., 2016; Yu et al., 2016; Xu et al., 2017; Yao et al., 2017), colorectal cancer (Huang et al., 2015; Wang et al., 2015; Guo et al., 2016; Xie et al., 2016; Hsiao et al., 2017; Weng et al., 2017; Zhu et al., 2017), gastric cancer (Li P. et al., 2015, 2017; Chen J. et al., 2017; Chen S. et al., 2017; Li W.H. et al., 2017; Sui et al., 2017; Zhao et al., 2018), gliomas (Barbagallo et al., 2016; Zheng et al., 2017), lung cancer (Wan et al., 2016; Zhang et al., 2020), breast cancer (Liu Z. et al., 2019), pancreatic ductal adenocarcinoma (Li et al., 2016), laryngeal cancer (Xuan et al., 2016), esophageal squamous cell carcinoma (Xia et al., 2016), basal cell carcinoma (Sand et al., 2016b), cutaneous squamous cell carcinoma (Sand et al., 2016a), ovarian carcinoma (Ahmed et al., 2016), cervical cancer (Wu et al., 2020), and nasopharyngeal carcinoma (NPC; Yang et al., 2020). During the oncogenesis process, circRNAs can serve as either oncogenes or tumor suppressors by inhibiting critical protein-coding and/or non-coding genes involved in oncogenesis. Currently, the study of detailed mechanisms through which circRNAs facilitate cancer development is still in its infancy. Nevertheless, it is apparent that the discovery of circRNAs has created a new dimension for us to elucidate the mechanism of carcinogenesis.

## Oncogenic Viruses, vcircRNAs, and Cancers

The family of human oncogenic viruses currently comprises seven members: Epstein-Barr virus (EBV), Kaposi’s sarcoma-associated herpesvirus (KSHV), human papillomavirus (HPV), Merkel cell polyomavirus (MCV), hepatitis B virus (HBV), hepatitis C virus (HCV), and human T-lymphotropic virus (also known as human T-cell leukemia virus type 1). Together, they account for 12–16% of total cancer cases in the world (White et al., 2014; Zapatka et al., 2020). Notably, only a small portion of infected persons develop cancers, which usually happens many years after initial infection (Mesri et al., 2014). It indicates that these oncogenic viruses are not fully oncogenic – they are necessary but not sufficient to cause cancers (Mesri et al., 2014). Indeed, for all these oncogenic viruses, cancer formation is believed to be a biological accident, but not an adaptation during their co-evolution with the hosts (Moore and Chang, 2017). After all, cancers are considered as evolutionarily dead-end events which put both hosts and viruses in danger (Moore and Chang, 2017). Viral transmission is the central point for the viral persistence and evolution. However, oncogenic viruses carried by these cancers are usually not permissive to produce sufficient infectious virions in order to support human-to-human transmission (Moore and Chang, 2017). The transmission of the oncogenic virus generally occurs among asymptomatic individuals. The dramatic increase of viral cancers in the immunocompromised individuals indicates that host immune surveillance plays a critical role in preventing viral cancer formation in the infected individuals (Moore and Chang, 2017).

So, why do these oncogenic viruses cause cancers? There is a continual battle between hosts and oncogenic viruses. Hosts have evolved various innate and adaptive immune strategies to eliminate viral infections. Meanwhile, oncogenic viruses have co-evolved strong immunoevasion and replication programs to counteract hosts’ defense systems (Mesri et al., 2014). During the process of immunoevasion and replication, some of the viral strategies such as proliferation and anti-apoptosis accidentally break host homeostasis and trigger cancer development. However, since the cancer development is a multifactorial and multistep process, additional co-factors such as chronic inflammation, environmental mutagens and immunosuppression are usually required for the formation of viral cancers (Mesri et al., 2014).

To date, increasing evidence has indicated that circRNAs play important roles in the regulation of RNA biogenesis and cell signaling. We are not surprised to see oncogenic viruses once again exploit these pathways to suit their needs of survival. Next, we have discussed a number of recently identified vcircRNAs and the possible relevance of these vcircRNA in virus infection and the development of virus-associated human malignancies.

## Epstein-Barr Virus

Epstein-Barr virus is also known as EBV or human herpesvirus 4. It is the first human tumor virus identified in 1964 and accounts for ∼200,000 new cancer cases annually (Rickinson and Kieff, 2007; Young et al., 2016; Shannon-Lowe and Rickinson, 2019). EBV is etiologically related to a number of human malignant diseases including Burkitt’s lymphoma (BL), Hodgkin’s disease (HD), extranodal nasal-type natural killer/T cell lymphoma (NKTL), posttransplant lymphoproliferative disease (PTLD) and lymphoma in AIDS and immunocompromised individuals, NPC, gastric carcinoma (GC), as well as lung cancers (LC) (de Sanjose et al., 2007; Rickinson and Kieff, 2007; Strong et al., 2013a, b; Young et al., 2016; Kheir et al., 2019; Shannon-Lowe and Rickinson, 2019).

Epstein-Barr virus belongs to the human γ-herpesvirus subfamily, in the same family with KSHV. There are two distinct stages in EBV’s life cycle: latency and lytic cycle (also known as lytic reactivation). After primary infection, EBV co-exists with its host permanently and exhibits a life-long latency. During the latency, the viral genome remains as an episomal chromosome and only a limited repertoire of viral genes are expressed. In response to certain physiological stimuli, the latent genome can be reactivated to express more than 100 viral genes and produce new infectious virions.

Infectious virions can be transmitted through various types of body fluids, but they are mainly spread through saliva exchange. There’s no anti-EBV vaccine currently available and it’s estimated that ∼90% of the world population permanently carries EBV (Rickinson and Kieff, 2007). To date, the detailed mechanism of EBV primary infection is still unclear. The current theory holds that EBV initially infects replication-permissive oropharyngeal epithelial cells in the host. The propagated infectious virions are then transmitted from the initial epithelial cells to the infiltrating B-cells in which the full set of viral latency transcripts are expressed (known as type III latency) (Table 1). The type III latency can subsequently activate B cells and cause a transient expansion of the EBV-infected B-cell pool. Majority of such B-cells will be eliminated by host immune surveillance. However, a sub-set of infected circulating B-cells can persist for the lifetime of the host. This is attributable to expression of no (type 0 latency) or only one viral protein (type I latency) (Table 1). It is believed that sporadic reactivation occurs in epithelial tissues as well as B cells differentiating into plasma cells (Laichalk and Thorley-Lawson, 2005; Sun and Thorley-Lawson, 2007), and the progeny infectious virions are secreted to the saliva and then transmitted to new hosts.

**TABLE 1:** EBV linear transcripts in different types of latency.

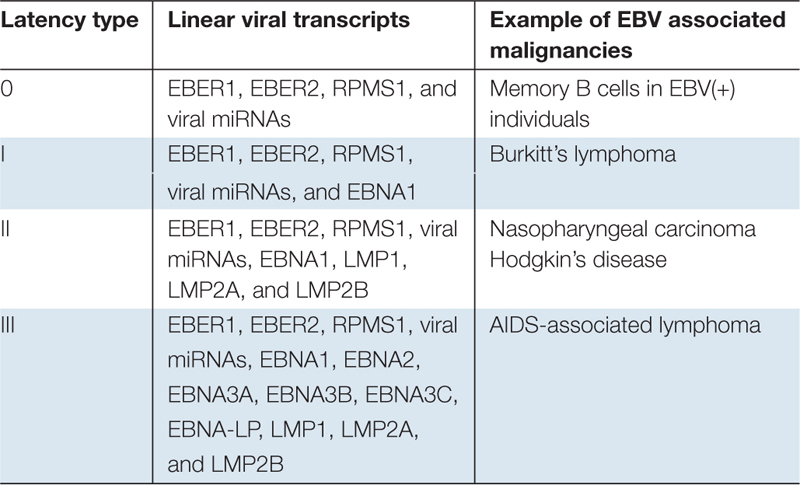

Previously, EBV has been shown to utilize non-coding linear RNAs such as miRNAs, long non-coding RNAs (lncRNAs), and small non-coding EBV-encoded RNAs (EBERs) to facilitate its life cycle and oncogenesis (Pfeffer et al., 2004; Cai et al., 2006; Hutzinger et al., 2009; Concha et al., 2012; Moss and Steitz, 2013; O’Grady et al., 2014; Cao et al., 2015a, b; O’Grady et al., 2016). Recently, increasing evidence has shown that EBV also encodes its own set of circRNAs to promote its survival. Since both the virus survival and tumor survival often share the same needs, the expression of EBV-encoded circRNAs can potentially contribute to the development of virus-associated cancers through the circRNA-dependent alteration of cancer pathways.

### Epstein-Barr Virus Encoded circRNAs

Human-to-human transmission is pivot to viral evolution, which requires establishment of long-term infection. Thus, powerful immunoevasion strategies are always needed for the viruses to accomplish their goal of persistence. Since circRNAs are largely non-immunogenic, vcircRNAs are viewed as ideal signal molecules to fulfill many virus needs by regulating host pathways without eliciting the adaptive/innate anti-viral immune responses. This may partially explain why more abundant vcircRNAs are observed in herpesviruses-infected cells. Furthermore, the circRNA biogenesis cascade is likely preferentially suited to DNA viruses. Hence, on one hand, the nuclear replication of these viruses allows them to easily access to many RBPs/splicesome components required for circRNA synthesis. On the other hand, the inherent bicistronic or polycistronic nature of viral transcripts also increases their chance of being backspliced by the splicesomes.

To date, vcircRNAs have been discovered in the transcriptomes of a number of human and non-human viruses including EBV, KSHV, HPV, HBV, MCV, rhesus macaque lymphocryptovirus (rLCV), murid herpesvirus 68 (MHV68), and rat polyomavirus 2 (RatPyV2) (Sekiba et al., 2018; Toptan et al., 2018; Ungerleider et al., 2018, 2019a; Zhao et al., 2019; Abere et al., 2020a, b). As shown in Table 2, the herpesvirus family holds the record for the highest number of vcircRNAs. Intriguingly, EBV is the first oncogenic human virus reported to express its own circRNAs and is also one of the top vcircRNA encoders.

**TABLE 2:** Summary of currently identified circRNAs encoded by human oncogenic viruses and their biological effects.

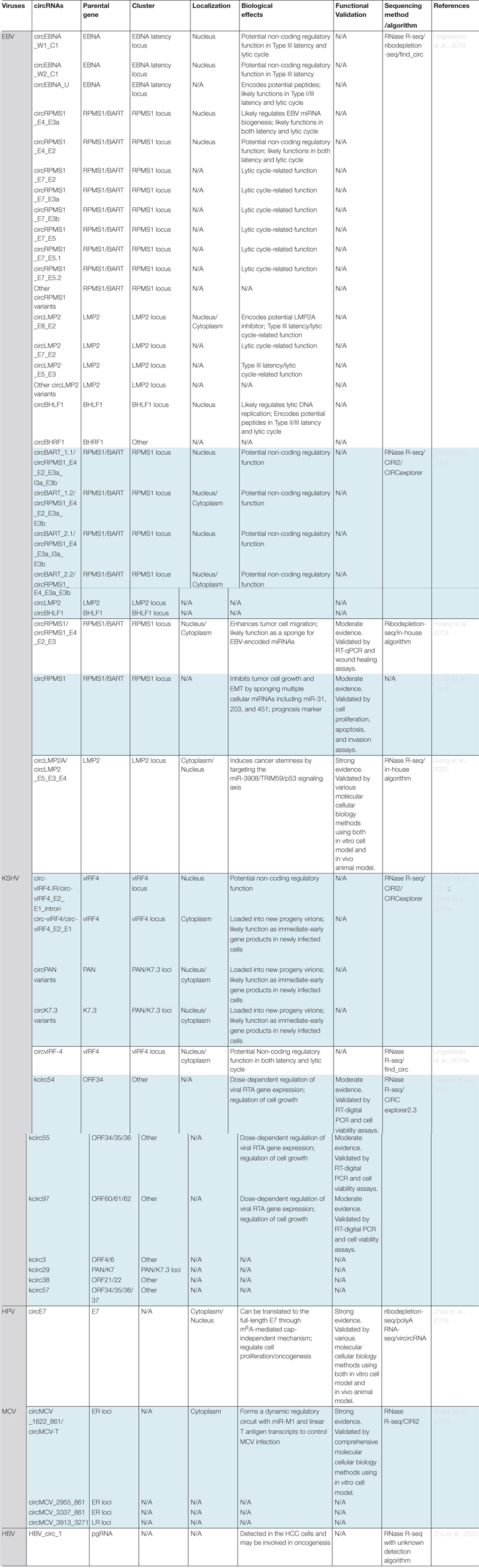

*N/A: Not applicable.*

Two research teams almost simultaneously utilized the RNase R-sequencing approach to assess vcircRNA expression in a variety of EBV infected cell models and patient tumor samples. In RNase R-sequencing, ribodepleted RNAs are treated with RNase R to remove the linear RNAs before library preparation. Thus, the sensitivity of circRNA detection is improved by enriching the circRNA species. Together, more than 30 EBV-encoded circRNAs (i.e., virally-encoded exonic circRNA and Exon-Intron circRNA) were identified, based on the unique backsplice junctions (Toptan et al., 2018; Ungerleider et al., 2018). Many of these vcircRNAs are expressed at comparable or higher levels to most cellular circRNAs in EBV-associated cancers including gastric cancer, NPC, BL, PTLD, and AIDS-associated lymphoma, indicating a potential functional significance of these vcircRNAs in EBV’s pathogenesis (Toptan et al., 2018; Ungerleider et al., 2018; Huang et al., 2019; Liu Q. et al., 2019; Qiao et al., 2019; Gong et al., 2020).

These identified vcircRNAs can be further grouped into 4 clusters. Below, we will discuss each cluster based on their parental gene loci.

#### RPMS1 Locus

A total of sixteen vcircRNAs were derived from the RPMS1 gene locus within the BamHI A rightward transcript (BART) region (Toptan et al., 2018; Ungerleider et al., 2018). The viral BART gene was initially discovered in EBV positive NPC cells (Hitt et al., 1989). Later on, BART transcripts were observed in both healthy viral carriers and patient with EBV-associated malignancies such as BL, HD, and gastric cancer (Deacon et al., 1993; Chiang et al., 1996; Sugiura et al., 1996; Tao et al., 1998; Webster-Cyriaque and Raab-Traub, 1998). In General, BART region is highly conserved among EBV strains except the B95-8 laboratory EBV strain has a 12-kb deletion of BART locus (Raab-Traub et al., 1980). BART transcripts are a set of highly diverse transcripts including long noncoding RNAs such as RPMS1 and more than 40 EBV viral miRNA spliced from the RPMS1 intronic sequences (Edwards et al., 2008; Yamamoto and Iwatsuki, 2012; Cao et al., 2015b). Both RPMS1 and viral miRNAs are involved in regulating cancer-related signaling pathways.

Four vcircRNAs derived from this locus were observed in all the examined latency types and virally infected cancer biopsies, but not in the B95-8 EBV infected cells due to a BART/RPMS1 locus deletion. Among them, circRPMS1_E4_E2_E3a_I3a_E3b (circBART_1.1) and circRPMS1_E4_E2_E3a_E3b (circBART_1.2) were formed by backsplicing of RPMS1 exon 4 to exon 2. Whereas, circRPMS1_E4_E3a_I3a_E3b (circBART_2.1), circRPMS1_E4_E3a_E3b (circBART_2.2) were formed by backsplicing of RPMS1 exon 4 to exon 3a. Interestingly, the intron-retained vcircRNAs circBART_1.1 and circBART_2.1 show nuclear distribution. In contrast, the exonic circBART_1.2 and circBART_2.2 are localized in both cytoplasm and nucleus.

#### EBNA Latency Locus

Three vcircRNAs are derived from the EBNA latency locus (Toptan et al., 2018; Ungerleider et al., 2018). Among them, circEBNA_W1_C1 and circEBNA_W2_C1 are formed by backsplicing of Bam HI W repeat W1 and W2 exons to the C1 exon. The expression of EBNA C1 exon is driven by the Bam HI C promoter (Cp) during the type III latency. Thus, these 2 vcircRNAs were mainly detected in type III latency cells with active Cp activity, but not in the Type I or II latency cells with epigenetically-silenced Cp. Meanwhile, circEBNA_U is generated by backsplicing of the Bam HI U fragment exon. Since the expression of EBNA U exon can be driven by all three EBNA promoters Cp, Wp, and Qp during both the latency and lytic cycles, the backsplicing of exon U was detected in all the examined type I and type III latency cells as well as cells harboring lytic reactivation.

#### LMP2 Locus

A total of eight vcircRNAs were derived from the LMP2 locus (Toptan et al., 2018; Ungerleider et al., 2019b). LMP2 is mainly a Type II/III latency protein that is expressed in a wide array of EBV associated B- and epithelial cell tumors where it likely contributes to the tumor phenotype (Yuan et al., 2006). Notably, the LMP2 protein is also expressed during the lytic stage, indicating a potential lytic function of these LMP2 products (Lu et al., 2006; Yuan et al., 2006; Concha et al., 2012). Furthermore, previous studies have shown that LMP2 locus undergoes extensive alternative splicing to generate various linear transcript isoforms during reactivation (Concha et al., 2012; O’Grady et al., 2014, 2016).

Among circLMP2s, high levels of circLMP2_E8_E2 were detected in reactivated Akata BL cells. This vcircRNA was generated by backsplicing of exon 8 to exon 2 of the LMP2 gene. Surprisingly, although Type III latency cells also constitutively express LMP2, circLMP2s were barely detected. It’s postulated that certain tissue specific splicing factors are likely involved in the circLMP2 biogenesis.

#### BHLF1 Locus

circBHLF1 is derived from the intra-exonic backsplicing of the BHLF1 gene (Toptan et al., 2018; Ungerleider et al., 2018). Increasing evidence has indicated that BHLF1 can be expressed in both latency and lytic cycles and may work as a non-coding gene for various EBV strains (Lin et al., 2010, 2013; Yetming et al., 2020). The expression level of circBHLF1 is largely consistent with the isogenic linear transcript and it can be detected in most of the latency and reactivation conditions examined.

### Regulation of EBV-Encoded circRNA Expression

Notably, although most of the vcircRNAs were encoded by the latency genes, many of these vcircRNAs can be detected in both latency and reactivation conditions. Nevertheless, higher levels of vcircRNAs were detected in the lytic reactivation. Liang et al. (2017) have shown that cell stress and associated depletion of spliceosomal components can increase production of circRNA by re-directing nascent RNAs into circRNA biogenesis pathways. Since reactivation elicits strong stress to the host transcription machinery, it’s possible that EBV might utilize similar strategy to promote vcircRNA production. However, the level of the backsplicing of cellular genes is not induced during reactivation (Ungerleider et al., 2018). It suggests that the expression bias is likely due to virus-related mechanisms but not simply resulting from attenuated linear canonical splicing induced by the reactivation-related stress responses. Among the candidate viral factors regulating the biogenesis/transportation of these vcircRNAs, BMLF1 likely attracts the most attentions. BMLF is a viral lytic gene and it is actively involved in splicing, polyadenylation, RNA transport, and translation by interacting with various host factors including splicing regulators (Semmes et al., 1998; Hiriart et al., 2005; Sergeant et al., 2008; Juillard et al., 2009, 2012; Mure et al., 2018). However, whether BMLF1 can indeed regulate the biogenesis of these vcircRNAs remains to be explored in the future.

### Function of EBV-Encoded circRNA Expression

#### RPMS1 Locus

Epstein-Barr virus circRNAs likely perform a wide spectrum of nuclear and cytoplasmic functions to facilitate viral pathogenesis. Given the universal expression of circRPMS1 in EBV-associated tumor tissues/cells as well as strong evolutionary conservation between EBV circRPMS1 and its rLCV homologue, it’s reasonable to hypothesize that circRPMS1 may play an important role to facilitate EBV pathogenesis and associated tumor development (Ungerleider et al., 2019a).

Indeed, Liu Q. et al. (2019) found higher levels of circRPMS1 in the metastatic NPC tissues collected from patients with a shorter overall survival. Further, the authors provided both *in vitro* and *in vivo* evidence showing that circRPMS1 likely functions as key oncogene to facilitate NPC development. By sequestering/sponging a number of host miRNAs including miR-203, miR-31 and miR-451, circRPMS1 can promote proliferation, apoptosis-resistance, and epithelial-mesenchymal-transition (EMT) of the tumor cells and thus enhance the oncogenic processes (Liu Q. et al., 2019).

In another work, Huang et al. utilized computational approaches to identify a number of human and EBV miRNAs that may be directly sequestered by circRPMS1 (Huang et al., 2019). Some potential miRNA candidates have been shown to negatively regulate tumor development (e.g., hsa-miR-28-5p) or modulate EBV lytic cycles (e.g., ebv-miR-BART20-5p). Moreover, enhanced expression of circRPMS1 in a gastric cancer cell model significantly downregulated host miRNA candidates and increased cell motilities. This evidence supports an oncogenic role of circRPMS1.

Recently, Qiao et al. (2019) did a more comprehensive bioinformatics analysis of miRNAs candidates sponged by the EBV circRNAs including circRPMS1. They predicted that these targeted miRNAs are likely involved in herpesvirus infection, cell cycle regulation and various oncogenic pathways.

Meanwhile, since the backsplicing sites for circRPMS1_E4_E3a_I3a_E3b (circBART_2.1) and circRPMS1_E4_E3a_E3b (circBART_2.2) are in close proximity to the intron encoding EBV miRNAs, it’s postulated that the backsplicing is likely to regulate these miRNA biosynthesis. Although circRPMS1 may still function as protein-coding RNAs in certain circumstances (e.g., in the *in vivo environment*), they are not associated with translated polysome fractions in the examined cells (Toptan et al., 2018).

#### EBNA Latency Locus

Given that both circEBNA_W1_C1 and circEBNA_W2_C1 are enriched in the nucleus, they are less likely to function as protein-coding transcripts. Further, the restricted expression of these vcircRNAs in Type III latency suggests that they may exhibit some latency-specific regulatory functions. For instance, they may be involved in the regulation of Cp activity and/or Cp-associated transcript diversity.

The evolutionary conservation of circEBNA_U between EBV and rLCV as well as similar expression of homologous rLCV_circEBNA_U in monkey lymphoma samples highly suggest a functional importance of this vcircRNA in viral pathogenesis (Ungerleider et al., 2019a). Interestingly, circEBNA_U contains an open reading frame (ORF) encoding a short 6-amino acid peptide. Moreover, ribosome profiling analysis showed possible translation initiation from this ORF (Bencun et al., 2018). With a potential IRES (internal ribosome entry site) translation initiation site, circEBNA_U may indeed encode some short peptides to regulate viral pathogenesis.

#### LMP2 Locus

Generally, LMP2 gene encodes two major isoforms, LMP2A and LMP2B. LMP2A is encoded by exons 1 to 9, and functions as a transmembrane signaling molecule (Longnecker and Kieff, 1990). In contrast, LMP2B is encoded by exons 2 to 9 (Longnecker and Kieff, 1990). Without the first exon encoding a cytoplasmic signaling domain, LMP2B mainly functions as a dominant-negative transmembrane inhibitor of LMP2A (Rovedo and Longnecker, 2007). Structure analysis shows that the circLMP2_E8_E2 likely carries all the coding sequence of the LMP2B. Since circLMP2_E8_E2 shows a significant cytoplasmic distribution, it’s likely that this vcircRNA could function as a novel isoform of LMP2B to regulate LMP2A’s function. In addition, the active expression of circLMP2_E8_E2 during the lytic cycle also indicates a potential role for this vcircRNA in reactivation.

Having analyzed the highly malignant SNU-4th cells isolated from the gastric cancer xenografts, Gong et al. provided evidence demonstrating that the EBV-encoded circLMP2A_E5_E3 likely functions as a miRNA sponge to induce and maintain cancer stemness by suppressing the anti-tumor effects of the miR-3908/TRIM59/p53 signaling, which is likely responsible for the metastasis and poor prognosis of EBV-associated gastric cancer (Gong et al., 2020).

#### BHLF1 Locus

Cell fraction analysis shows that the circBHLF1 is mainly localized in the nucleus, indicating a nuclear function of this vcircRNA. Interestingly, previous studies have shown that BHLF1 linear transcript is associated with the lytic replication complex and promotes lytic viral DNA replication (Rennekamp and Lieberman, 2011). Thus, circBHLF1 may similarly interact with the proximal oriLyt DNA sequences and regulate viral DNA replication. The expression of circBHLF1 during the latency indicates that this vcircRNA may exhibit certain latency-related function as well.

circBHLF1 also carries an ORF encoding a putative 200 amino acid peptide. Evidence of translation initiation at this genomic location was reported previously (Bencun et al., 2018). However, it still remains unclear if the low abundance of circBHLF in the cytoplasm can produce this putative peptide, or if the product is functional.

## Kaposi’s Sarcoma-Associated Herpesvirus

Kaposi’s sarcoma-associated herpesvirus is also known as human herpesvirus 8 and is causatively associated with a number of human malignant diseases including Kaposi’s sarcoma (KS), primary effusion lymphoma (PEL), and multicentric Castleman’s disease (MCD) (Chang et al., 1994; Cesarman et al., 1995, 1996; Soulier et al., 1995). As a human γ-herpesvirus, KSHV also has two distinct stages, latency and lytic phase in its life cycle. During the latency, relatively restricted sets of viral genes are expressed in order to minimize the mounted host adaptive immunity (Staskus et al., 1997; Katano et al., 2000; Parravicini et al., 2000; Hosseinipour et al., 2014). In the lytic cycle, more than 90 viral genes are expressed to facilitate the production of new infectious virions.

Like EBV, KSHV encodes its own non-coding linear RNAs to facilitate their needs for long-term persistence and transmission. At least 25 mature miRNAs have been identified in KSHV. This cluster of miRNAs is derived from a viral latency-associated genomic region and plays critical roles to support viral latency and associated oncogenesis (Cai et al., 2005; Pfeffer et al., 2005; Samols et al., 2005; Hansen et al., 2010; Lei et al., 2010a, b; Qin et al., 2010; Umbach and Cullen, 2010; Gottwein et al., 2011).

KSHV also encodes a number of lncRNAs including PAN (polyadenylated nuclear) RNA, K7.3, T3.0, T1.2, and ALT (antisense-to-latency transcript) (Staskus et al., 1997; Sarid et al., 1998; Wang et al., 2004; Taylor et al., 2005; Chandriani et al., 2010; Majerciak et al., 2013; Arias et al., 2014; Rossetto and Pari, 2014; Schifano et al., 2017). Most of these viral lncRNAs show increased expression during the lytic cycle and PAN RNA accounts for more than 80% of the viral transcripts during viral reactivation (Rossetto and Pari, 2014). Further, PAN RNA is also the most thoroughly studied KSHV lncRNA and it’s involved in transcriptional and epigenetic regulation of host and viral gene expression (Rossetto and Pari, 2011, 2012; Rossetto et al., 2013). For instance, PAN facilitates late lytic gene expression in the nucleus and PAN may also modulate the host immune response to facilitate viral infection (Borah et al., 2011; Rossetto and Pari, 2011, 2014; Rossetto et al., 2013; Campbell et al., 2014a, b). Intriguingly, cytoplasmic PAN transcripts can be actively loaded into KSHV virions and likely function as immediate early RNA in the newly infected cells (Bechtel et al., 2005). K7.3 RNA is derived from the opposite strand to PAN. K7.3 RNA is also increased during lytic cycle and it’s predicted to exhibit some non-coding function (Dresang et al., 2011; Schifano et al., 2017).

### Kaposi’s Sarcoma-Associated Herpesvirus Encoded circRNAs

Recently, several groups have reported that KSHV encoded a repertoire of circRNAs in virally-infected cell lines and clinical specimens including primary KSHV-associated tumor tissues and blood samples (Tagawa et al., 2018; Toptan et al., 2018; Ungerleider et al., 2019a; Abere et al., 2020a). These vcircRNAs are mainly derived from 2 genomic loci. In addition, a number of low-abundant vcircRNAs (such as kcirc3, kcirc38, kcirc54, kcirc55, kcirc57, and kcirc97) derived from several other genomic regions were also detected in certain cell model systems (Tagawa et al., 2018).

#### PAN/K7.3 Loci

More than 100 low-abundance vcircRNAs are derived from the PAN/K7.3 loci (Tagawa et al., 2018; Toptan et al., 2018; Ungerleider et al., 2019a; Abere et al., 2020a). These vcircRNAs can be further categorized into two distinct clusters: circPAN and circK7.3 based on their parental sense (PAN) as well as antisense (K7.3) transcripts, respectively. Although the level of individual vcircRNA within each cluster is low, the accumulated abundance of the circPAN and circK7.3 is very high and they become a major component of the KSHV circRNAome. Both circPAN and circK7.3 were detected in the analyzed PEL cell lines regardless of KSHV life cycle, as well as KSHV-associated tumor tissues (e.g., KS and MCD) and blood samples. Further, both circPAN and circK7.3 are distributed in both cytoplasm and nucleus.

#### vIRF Locus

circ-vIRF4s are derived from the vIRF4 (viral interferon regulatory factor 4)/K10 gene locus. The parental gene vIRF4 together with three other vIRFs namely vIRF1, vIRF2, and vIRF3 locate in a 10 kb genomic segment. The vIRF4 is homologous to cellular IRF4 and can inhibit the cellular IRF4 and it’s also involved in cell cycle control and oncogenic process (Lee et al., 2009; Jacobs and Damania, 2011).

circ-vIRF4s are generated by backsplicing of exon 2 to exon 1. Two isoforms are observed. circ-vIRF4 is a 530 bp exonic circRNA, while circ-vIRF4.IR is a 632 bp Exon-Intron circRNA. Differential subcellular distributions were observed for the two circ-IRF4 isoforms. The intron retention form (circ-vIRF4.IR) is predominantly enriched in the nucleus. The exon-only form (circ-vIRF4) is mainly located in the cytoplasm. RNA decay analysis showed that the half-life of circ-vIRF4 (∼5 h) is significantly longer than the one of linear vIRF4 mRNA (∼2 h). circ-vIRF4s can be detected in PEL cell lines, KSHV-associated tumor tissues (e.g., KS and MCD) and blood samples.

### Regulation of KSHV-Encoded circRNA Expression

The overlapping nature of circPAN and circK7.3 makes the characterization work more challenging. None of those backsplicing junctions are utilized to splice the known linear RNA transcripts from the PAN/K7.3 loci. To date, the mechanism for such hypervariable and bidirectional synthesis of circPAN/K7.3 remains unclear. Notably, circPAN and circK7.3 can be detected in both latency and lytic reactivation. After lytic induction, the levels of circPAN and circK7.3 are increased in parallel with the linear PAN and K7.3 transcripts. Thus, the synthesis of circPAN/K7.3 is likely at least partially regulated by the transcription initiation of their parental genes.

circ-vIRF4s are constantly expressed in both latency and lytic cycle. Although the parental gene vIRF4 is induced after lytic induction, the levels of circ-vIRF4s are barely increased or even decreased in some cell models. In certain cell models (e.g., BCP-1 and BBG1), higher levels of circ-IRF4s were observed than the isogenic linear transcript. This indicates that the observed latent circ-vIRF4s are regulated by an independent mechanism compared to the biosynthesis of linear vIRF4 transcript. For instance, the expression of circ-vIRF4s may be regulated by an unrecognized latency promoter.

### Function of KSHV-Encoded circRNAs

To date, the roles of these vcircRNAs in KSHV’s pathogenesis are still largely unclear. However, given the important roles of the parental genes of these vcircRNAs (as discussed earlier) and their (constitutive) expression profiles, it’s postulated that these non-polysome-associated vcircRNAs are most likely to function as non-coding regulatory RNAs.

Intriguingly, circPAN, circK7.3, and cytoplasmic circ-vIRF4 (but not the nucleus circ-vIRF4.IR) can be actively loaded into the KSHV infectious virions. It’s hypothesized that these vcircRNAs, just like viral tegument proteins carried by the progeny virions (Sathish et al., 2012), may function as immediate-early gene products and host-immune modulators to help establish the infection in the newly infected cells.

Recently, Tagawa et al. showed that enhanced expression of some individual vcircRNAs such as kcirc54, kcirc55, and kcirc97 suppressed the transcription of viral lytic gene RTA and also induced an altered growth behaviors. This suggests a potential role of these vcircRNAs in KSHV pathogenesis (Tagawa et al., 2018).

## Human Papillomavirus

Human papillomaviruses are small non-enveloped, double-stranded DNA viruses (Brentjens et al., 2002; Roden and Stern, 2018). To date, more than 200 types of HPV have been identified and they show epitheliotropic and mainly infect mucosal and cutaneous epithelia (Roden and Stern, 2018). Based on the clinical consequence of the viral infection, HPV can be classified as low-risk HPVs (LR-HPVs) that generally cause benign lesions such as warts, as well as high-risk HPVs (HR-HPVs) that can transform the infected cells and promote cancer development (Roden and Stern, 2018). Among HR-HPVs, HPV16, and HPV18 are the most prevalent high-risk types and are etiologically associated with a large number of human cancers that occur in the cervical, anogenital, and head and neck regions. The recent development and implementation of anti-HPV vaccines successfully help to reduce new infection caused by several HR-HPV and LR-HPV types, but unfortunately do not have any effect on the existing persistent infection (Roden and Stern, 2018). Since the development of HPV-associated cancers is a chronic process, it is predicted that the HPV-associated malignancies will continuously cause significant medical problems in the next 50 years at least.

Human papillomavirus has a small ∼8 kb DNA genome which carries both the early (E) gene loci and late (L) gene loci (Brentjens et al., 2002). The E loci are regulated by the early-promoter and they encode proteins (E1–E7) required for viral replication. Meanwhile, the late-promoter-regulated L loci encode the structural proteins (L1–L2) for progeny virion assembly (Brentjens et al., 2002). HR-HPV E6 and E7 are the two most characterized viral oncogenes. They can inhibit major tumor suppressor proteins such as p53 and Rb and promote cancer development (Brentjens et al., 2002). So far, HPV has not been reported to encode any linear non-coding transcripts such as lncRNA and miRNA.

### Human Papillomavirus Encoded circRNAs

Recently, a number of HPV-encoded circRNAs have been detected in HR-HPV-infected cell model systems and clinical cancer samples (Chamseddin et al., 2019; Zhao et al., 2019). These vcircRNAs are generated by backsplicing of various splice sites located in both the E and L loci. Among these vcircRNAs, the most abundant form is known as circE7 which accounts for ∼1–3% of total E7 transcript and it is first identified in the HPV16 infected cells and cancer tissues. Analogous circE7 has been detected in other types of HR-HPVs including HPV18, 31, 33, 35, 45, and 58. This 472 bp vcircRNA is generated by joining a downstream splice donor in E1 gene to an upstream splice acceptor in E6 gene. circE7 also carries the entire ORF of E7.

### Regulation of HPV-Encoded circRNA Expression

Current evidence shows that the biogenesis of circE7 is likely partially regulated by its m^6^A (N^6^-methyladenosine) sites (Zhao et al., 2019). Indeed, mutation of circE7’s m^6^A sites leads to a decreased level of circE7 transcripts, indicating that these sites may be targeted by splicing factors required for circRNA biogenesis.

### Function of HPV-Encoded circRNA Expression

The identification of multiple candidate miRNA binding sites on circE7 suggests that this vcircRNA may function as a miRNA sponge. Further, knockdown of HPV16 circE7 leads to an increased level of the linear E6/E7 transcripts, suggesting that circE7 may regulate the biogenesis of its isogenic transcripts by competing for their shared splice sites.

Besides its potential non-coding function, circE7 is likely to function as a protein-coding RNA, since it is enriched in the cytoplasm and also associated with translated polysome fractions. circE7 has been shown to produce a full length E7 protein in various cell models. The translation is apparently initiated through an m^6^A (N^6^-methyladenosine)-mediated cap-independent mechanism. HPV16 circ-E7-mediated expression of E7 protein has been shown to regulate cell proliferation, anchorage-independent cell growth, as well as oncogenesis in both *in vitro* cell models and *in vivo* tumor xenografts. Further, the expression of circ-E7 in virally infected non-cancer cells indicates that this vcircRNA may also play a role in viral infection cycle.

## Merkel Cell Polyomavirus

Merkel cell polyomavirus is causally associated with a majority of the Merkel cell carcinoma, an aggressive skin cancer mainly affecting elderly and immunocompromised individuals (Feng et al., 2008; Shuda et al., 2008). MCV is a nonenveloped virus with a circular double-stranded DNA genome and belongs to the human polyomavirus family (Feng et al., 2008; Shuda et al., 2008). In the infected cells, the MCV genome can remain either as a free episomal DNA undergoing active replication, or as a replication-deficient form after integrating into the host genome (Feng et al., 2008; Shuda et al., 2008). It is believed that genome integration plays a critical role in MCV-mediated oncogenesis (Feng et al., 2008; Shuda et al., 2008). Its relatively small DNA genome can be simply divided into two gene loci, the early region (ER) and late region (LR). Generally, the ER-encoded gene products such as large T antigen (LT) are involved in the regulation of viral replication and oncogenesis. For instance, the LT can directly target tumor suppressor protein Rb. Meanwhile, it can bind to the viral origin of replication and initiate viral genome replication with its intrinsic helicase/ATPase activity (Feng et al., 2008; Shuda et al., 2008). In contrast, the LR loci encode viral structural proteins such as VP1 and VP2 required for new virion production (Tolstov et al., 2009).

Besides viral protein products, MCV also encodes two miRNAs, namely MCV-miR-M1-5p and MCV-miR-M1-3p, which are derived from the pri-miRNA transcript synthesized from the negative strand of the ER (Seo et al., 2009). These 22 nucleotide viral miRNAs have been shown to inhibit MCV lytic replication by knocking-down the LT transcripts in order to facilitate maintenance of the episomal genome (Seo et al., 2009; Theiss et al., 2015).

### Merkel Cell Polyomavirus Encoded circRNAs

Recently, MCV has been shown to encode four circRNAs (Abere et al., 2020b). Among them, three vcircRNA are derived from the ER loci and one is encoded by the LR loci. circMCV-T (circMCV_1622_861) is the most abundant one and accounts for more than 70% of the total vcircRNA population. circMCV-T is a 762 bp vcircRNA carrying the entire exon II of the T antigen. It’s generated by backsplicing of the downstream 5′ splice donor (nt 1622) to the upstream 3′ splice acceptor (nt 861). These splice sites are also required for the splicing of all the linear T antigen isoforms including LT, small T antigen (sT), 57,000-molecular-weight T antigen (57 kT), and alternative LT open reading frame (ALTO) transcripts. circMCV_2955_861 and circMCV_3337_861 use the same 3′ splice acceptor sites but different 5′ splice donor sites at nt 2955 and 3337, respectively. circMCV_3913_3271 shows lowest abundance and is derived from the LR loci. To date, among these vcircRNAs, only the most abundant circMCV-T was further characterized.

### Regulation of MCV-Encoded circRNA Expression

The circMCV-T transcripts are detected mainly in the cells harboring actively replicating episomal viral genomes, but rarely in the cells carrying more dormant integrated viral genome. This suggests that the biogenesis of circMCV-T is at least partially regulated by the transcription activity of its parental ER loci.

### Function of MCV-Encoded circRNAs

A circMCV-T homologue, namely circRatPyV2_4112_4468 was recently detected in the RatPyV2 infected cells (Abere et al., 2020b). The evolution conservation of circMCV-T further indicates a functional significance of this RNA molecule. Increasing evidence has shown that the MCV-encoded transcripts including circMCV-T, miR-M1, and linear T antigen transcripts form a dynamic regulatory circuit to control MCV’s life cycle. Due to a 100% sequence complementarity between miR-M1 and linear T antigen mRNAs, the miRNA-mRNA interaction can knock down linear T antigen transcripts and inhibit viral DNA replication. The presence of the new player circMCV-T further increases the complexity of the network. Due to a 100% sequence complementarity between miR-M1 and circMCV-T, the reciprocal interaction between circMCV-T and miR-M1 can lead to two scenarios. In the first scenario, miR-M1 can decrease the stability of circMCV-T by linearizing it and thus reduce the genome replication. In the second, circMCV-T can increase the level of linear T antigen transcripts and facilitate viral replication by sequestering miR-M1. Hence, the overall effects will be determined by the relatively amount of miR-M1 and circMCV-T: a condition of high circMCV-T and low miR-M1 will favor active MCV replication. Conversely, a low circMCV-T and high miR-M1 condition will suppress active viral replication and promote a long-term persistent infection.

Besides the non-coding function, the circMCV-T transcript may potentially function as a protein-coding RNA. The in situ staining data show that circMCV-T is predominantly located in the cytoplasm. It also carries the entire exon 2 of T-antigen as well as the start codon for the ALTO ORF. However, some data show that circMCV-T transcripts are not associated with any translated polysomes in certain cell models.

## Hepatitis B Virus

Hepatitis B virus is a small oncogenic human virus closely associated with liver cirrhosis and HCC (Torresi et al., 2019). HBV has a 3.2 kb partially double-stranded relaxed circular DNA (rcDNA) genome. In the infected cells, a covalently closed circular DNA (cccDNA) genome is formed and remains as a persistent episomal genome. The cccDNA is used as templates to generate key transcripts including the pregenomic RNA (pgRNA) transcript for viral replication.

### Hepatitis B Virus Encoded circRNA

An HBV-encoded circRNA has been recently identified in both HBV-infected cell models and HCC tissues (Sekiba et al., 2018; Zhu et al., 2020). Zhu et al. (2020) has shown that this 2.5 kb vcircRNA, namely HBV_circ_1 is derived by the intronless pgRNA. Surprisingly, instead of utilizing the backsplicing, it is believed that this vcircRNA is generated by a mechanism involving the homologous recombination of the inverted repeat sequences at both 3′ and 5′ ends of the pgRNA (Zhu et al., 2020). In addition, HBV_circ_1 is predominantly located in the cytoplasm (Zhu et al., 2020).

### Regulation of HBV-Encoded circRNA Expression and Potential Function

Sekiba et al. (2018) showed that knockdown of an RBP DXH9 increases the levels of HBV_circ_1. It is proposed that the expression of HBV_circ_1 can be negatively regulated by the direct interaction between DHX9 and the inverted repeat regions of the pgRNA. To date, the biological function of HBV_circ_1 remains unclear.

## Potential Clinical Application of vcircRNAs

To date, detection of viral genomic material has been widely implemented in the clinical practice. For instance, circulating EBV DNA has been used as a good biomarker for the diagnosis and prognosis of EBV-associated NPC (Lo et al., 1999; Leung et al., 2006; Chan et al., 2017). Recently, circRNAs have also received increasing attention for its potential clinical applications. These highly stable vcircRNAs that exist in various types of body fluids and tissues may serve as good diagnostic and prognostic markers for many virally associated diseases.

Current evidence showed that vcircRNAs might be superior to the current RNA diagnostic marker. For EBV, EBERs are used as a gold standard for diagnosis of EBV infection (Ambinder and Mann, 1994). However, data showed that circRPMS1 were detected in certain PTLD tissues carrying reasonable number of EBV genomes, where EBER cannot be detected, indicating potential false-negative results coming from the EBER diagnostic test (Toptan et al., 2018). For KSHV, circPAN/K7.3 and circ-vIRF4 showed a higher detection rate than the linear viral transcript LANA (latency-associated nuclear antigen) in the analyzed primary KS tumor and associated blood samples (Abere et al., 2020a). Lastly, in the case of HPV, PCR-based detection of HR-HPV has been used for cervical cancer screening. The current data show that circE7 can be detected in fixed archive tissue samples as well (Chamseddin et al., 2019). It is warranted to investigate if circE7 may serve as a promising biomarker for HPV infection and HPV-related cancers.

## Challenges and Future Directions

Due to the recent development of circRNA enrichment and high-throughput detection technologies, we are now in a rapidly growing era of vcircRNA research. Numerous novel vcircRNAs have been identified in human oncogenic viruses, and they are no longer viewed as insignificant splicing noise. Instead, vcircRNAs are now seen for what they are: an important new class of regulatory molecules in viral infection cycle and associated malignant diseases. The addition of this new member has created an entirely new layer of complexity of the viral pathogenic mechanism.

Nevertheless, the vcircRNA research is in its nascent stage where many puzzles remain to be solved. Currently, the underlying mechanism for the biogenesis of vcircRNAs is still largely unclear. It’s postulated that, in addition to the general splicing machinery, both tissue-specific host factors and viral factors are likely to be involved in the formation of vcircRNAs. For instance, EBV circLMP2_E8_E2 can be detected in reactivated BL cells, but not in the type III latency cell lines that constitutively express parental linear LMP2A transcripts. It indicates that some tissue-specific factors may regulate the backsplicing by targeting the *cis* regulatory elements in the primary LMP2 transcript (Ungerleider et al., 2018). Meanwhile, EBV viral lytic gene BMLF1 is known to play critical roles in the maturation of viral linear transcripts and likely contribute to the backsplicing process. Future research is warranted to elucidate the detailed mechanism of vcircRNA synthesis.

Notably, within the family of human oncogenic viruses, it is still unclear if HCV or human T-lymphotropic virus (human T-cell leukemia virus type 1 or HTLV-1) encodes any vcircRNAs. Future research is surely warranted to investigate if any vcircRNAs are present in the HCV or HTLV-1-positive biospecimen. The RNase R-sequencing will be an ideal approach to evaluate the vcircRNAome in a high-throughput and unbiased manner.

Among all the human oncogenic viruses investigated so far, herpesviruses are undoubtedly the largest producer of vcircRNAs and account for >85% of the identified vcircRNAs. This expression bias may be partially attributable to herpesviruses’ unique bi-phasic life cycles: they can exist in both latent and lytic phases. Particularly during the latency, to escape the host immune surveillance, herpesviruses cannot express antigenic viral proteins, while they still need to regulate themselves and host environment. Hence, expression of non-coding RNAs which lack immunogenicity is likely an ideal strategy for the viruses to regulate themselves and the host environment when an immunologically transparent state is required. Increasing evidence has shown that vcircRNAs are less immunogenic, compared to linear transcripts. Chen et al. reported that unmodified exogenous circRNAs (without any nucleoside modifications) are recognized as “nonself” RNAs by the cellular sensors such as RIG-I and thus trigger the innate immune signaling. However, the circRNAs spliced by endogenous human spliceosomes are viewed as “self” RNAs and lose the immunogenicity, due to the N^6^-methyladenosine (m^6^A) modification during the biogenesis (Chen Y.G. et al., 2017; Chen et al., 2019). Based on the high prevalence of m^6^A modification of viral transcripts and evidence that vcircRNAs are largely generated by the same host splicesomes used to synthesize “self” circRNAs, it is reasonably to believe that vcircRNAs are non-immunogenic (Wu F. et al., 2019; Lu et al., 2020). Notably, Wesselhoeft et al. recently reported a discordant observation that immunogenicity of circRNA is independent of m^6^A modification. They found that unmodified exogenous circRNAs are also non-immunogenic and can avoid antiviral defense induction upon cellular entry by dodging immune sensors such as RIG-I and Toll-like receptor (Wesselhoeft et al., 2019). Although this new discovery still supports the notion that vcircRNAs are non-immunogenic, more efforts are warranted to investigate the discrepant data on the mechanism of immunoevasion of foreign circRNAs.

To date, only a handful of identified vcircRNAs are functionally investigated. A majority of these characterized vcircRNAs are enriched in the cytoplasm and are proposed to function as either miRNA sponges or protein-coding transcripts to regulate viral pathogenesis. These conclusions are largely based on the data from the *in vitro* cell models. However, the functional impacts of the large number of nuclear vcircRNAs have not yet been reported. These nuclear vcircRNAs such as EBV circRPMS1_E4_E2_E3a_I3a_E3b (circBART_1.1) and circRPMS1_E4_E3a_I3a_E3b (circBART_2.1) generally carry retained-introns from their parental genes. Due to their high abundance and nuclear distribution, they may play important regulatory roles such as modulating the levels of their isogenic transcripts by competing the splice sites and limited splicing factors. Further investigation is surely warranted.

The detailed transcript structures of these vcircRNA candidates are largely unclear. The size and sequence of most of these vcircRNAs are predicted simply based on their backsplicing sites. In-depth sequencing-based assembly and validation of the vcircRNAs is surely warranted, which is essential for the subsequent characterization work to understand the biogenesis and function of each vcircRNA isoform. It will be interesting to see if some new computation pipelines designed for reconstruction of full-length circRNA can be successfully implemented in vcircRNA research (Wu J. et al., 2019; Zheng et al., 2019).

To date, all the reported vcircRNAomes of these human oncogenic viruses are generated by RNA-seq analyses of bulk cell cultures and/or clinical tissues. Due to the inevitable heterogeneity of tissue/cell culture, the vcircRNAomes are likely different at the single-cell level. Thus, the expression and function of these vcircRNAs might vary among single cells. Implementation of single-cell sequencing technologies which are compatible with circRNAs might help us better decipher the biogenesis mechanism and function of vcircRNAs (Fan et al., 2015).

Our understanding of the biogenesis mechanism and function of these vcircRNAs are largely based on the data derived from *in vitro* cell cultures. These data need to be further validated and examined in more physiological relevant *in vivo* model systems such as animal models and clinical biospecimens.

The altered expression of vcircRNAs in infected host cells and diseased tissues as well as the long half-life of vcircRNAs make them potential biomarkers for clinical diagnosis of viral infection and prognosis of disease outcomes. Some of the oncogenic vcircRNAs such as circLMP2A might serve as potential targets for precision therapy. Given the successful implementation of mRNA-based vaccines to prevent SARS-CoV-2 infection, future highly stable circRNA-based vaccines might become a new tool to better prevent human oncogenic virus infection and related diseases.

The discoveries of viral miRNAs and lncRNAs and their roles have dramatically increased our knowledge of how oncogenic viruses interact with hosts and facilitate cancer development. Just like in the early years of viral miRNA and lncRNA research, the vcircRNA research has now started gaining increased attention and becoming an important topic in the viral oncology field. With further improvement of the circRNA analytical techniques, we expect that more vcircRNAs will be identified and successfully characterized in the coming years. These developments will help us better understand the roles of human oncogenic viruses in their associated cancers and improve patient prognosis in the fight against these deadly diseases.

## Author Contributions

ZL, JA, DB, and RA contributed to the conceptualization, drafting, and revision of the manuscript. AN, JK, and LL contributed to the conceptualization and manuscript revision. All authors contributed to the article and approved the submitted version.

## Conflict of Interest

The authors declare that the research was conducted in the absence of any commercial or financial relationships that could be construed as a potential conflict of interest.

## References

[B1] Abdelmohsen K. Panda A. C. Munk R. Grammatikakis I. Dudekula D. B. De S. (2017). Identification of HuR target circular RNAs uncovers suppression of PABPN1 translation by CircPABPN1. *RNA Biol.* 14 361–369. 10.1080/15476286.2017.1279788 28080204 PMC5367248

[B2] Abere B. Li J. Zhou H. Toptan T. Moore P. S. Chang Y. (2020a). Kaposi’s sarcoma-associated herpesvirus-encoded circrnas are expressed in infected tumor tissues and are incorporated into virions. *mBio* 11:e3027-19. 10.1128/mBio.03027-19 PMC6946807 31911496

[B3] Abere B. Zhou H. Li J. Cao S. Toptan T. Grundhoff A. (2020b). Merkel cell polyomavirus encodes circular RNAs (circRNAs) enabling a dynamic circRNA/microRNA/mRNA regulatory network. *mBio* 11:e3059-20. 10.1128/mBio.03059-20 PMC7773998 33323517

[B4] Ahmed I. Karedath T. Andrews S. S. Al-Azwani I. K. Mohamoud Y. A. Querleu D. (2016). Altered expression pattern of circular RNAs in primary and metastatic sites of epithelial ovarian carcinoma. *Oncotarget* 7 36366–36381. 10.18632/oncotarget.8917 27119352 PMC5095006

[B5] Aktas T. Avsar Ilik I. Maticzka D. Bhardwaj V. Pessoa Rodrigues C. Mittler G. (2017). DHX9 suppresses RNA processing defects originating from the Alu invasion of the human genome. *Nature* 544 115–119. 10.1038/nature21715 28355180

[B6] Ambinder R. F. Mann R. B. (1994). Epstein-barr-encoded RNA in situ hybridization: diagnostic applications. *Hum. Pathol.* 25 602–605. 10.1016/0046-8177(94)90227-5 8013952

[B7] Arias C. Weisburd B. Stern-Ginossar N. Mercier A. Madrid A. S. Bellare P. (2014). KSHV 2.0: a comprehensive annotation of the Kaposi’s sarcoma-associated herpesvirus genome using next-generation sequencing reveals novel genomic and functional features. *PLoS Pathog.* 10:e1003847. 10.1371/journal.ppat.1003847 24453964 PMC3894221

[B8] Arnberg A. C. Van Ommen G. J. Grivell L. A. Van Bruggen E. F. Borst P. (1980). Some yeast mitochondrial RNAs are circular. *Cell* 19 313–319. 10.1016/0092-8674(80)90505-x 6986989

[B9] Ashwal-Fluss R. Meyer M. Pamudurti N. R. Ivanov A. Bartok O. Hanan M. (2014). circRNA biogenesis competes with pre-mRNA splicing. *Mol. Cell.* 56 55–66. 10.1016/j.molcel.2014.08.019 25242144

[B10] Barbagallo D. Condorelli A. Ragusa M. Salito L. Sammito M. Banelli B. (2016). Dysregulated miR-671-5p / CDR1-AS / CDR1 / VSNL1 axis is involved in glioblastoma multiforme. *Oncotarget* 7 4746–4759. 10.18632/oncotarget.6621 26683098 PMC4826240

[B11] Bechtel J. Grundhoff A. Ganem D. (2005). RNAs in the virion of Kaposi’s sarcoma-associated herpesvirus. *J. Virol.* 79 10138–10146. 16051806 10.1128/JVI.79.16.10138-10146.2005 PMC1182685

[B12] Bencun M. Klinke O. Hotz-Wagenblatt A. Klaus S. Tsai M. H. Poirey R. (2018). Translational profiling of B cells infected with the Epstein-Barr virus reveals 5’ leader ribosome recruitment through upstream open reading frames. *Nucleic Acids Res.* 46 2802–2819. 10.1093/nar/gky129 29529302 PMC5887285

[B13] Borah S. Darricarrere N. Darnell A. Myoung J. Steitz J. A. (2011). A viral nuclear noncoding RNA binds re-localized poly(A) binding protein and is required for late KSHV gene expression. *PLoS Pathog.* 7:e1002300. 10.1371/journal.ppat.1002300 22022268 PMC3192849

[B14] Brentjens M. H. Yeung-Yue K. A. Lee P. C. Tyring S. K. (2002). Human papillomavirus: a review. *Dermatol. Clin.* 20 315–331. 12120445 10.1016/s0733-8635(01)00028-6

[B15] Cai X. Lu S. Zhang Z. Gonzalez C. M. Damania B. Cullen B. R. (2005). Kaposi’s sarcoma-associated herpesvirus expresses an array of viral microRNAs in latently infected cells. *Proc. Natl. Acad. Sci. U.S.A.* 102 5570–5575. 10.1073/pnas.0408192102 15800047 PMC556237

[B16] Cai X. Schafer A. Lu S. Bilello J. P. Desrosiers R. C. Edwards R. (2006). Epstein-Barr virus microRNAs are evolutionarily conserved and differentially expressed. *PLoS Pathog.* 2:e23. 10.1371/journal.ppat.0020023 16557291 PMC1409806

[B17] Campbell M. Kim K. Y. Chang P. C. Huerta S. Shevchenko B. Wang D. H. (2014a). A lytic viral long noncoding RNA modulates the function of a latent protein. *J. Virol.* 88 1843–1848. 10.1128/jvi.03251-13 24257619 PMC3911622

[B18] Campbell M. Kung H. J. Izumiya Y. (2014b). Long non-coding RNA and epigenetic gene regulation of KSHV. *Viruses* 6 4165–4177. 10.3390/v6114165 25375882 PMC4246214

[B19] Cao S. Moss W. O’grady T. Concha M. Strong M. J. Wang X. (2015a). New noncoding lytic transcripts derived from the epstein-barr virus latency origin of replication, oriP, are hyperedited, bind the paraspeckle protein, NONO/p54nrb, and support viral lytic transcription. *J. Virol.* 89 7120–7132. 10.1128/jvi.00608-15 25926645 PMC4473578

[B20] Cao S. Strong M. J. Wang X. Moss W. N. Concha M. Lin Z. (2015b). High-throughput RNA sequencing-based virome analysis of 50 lymphoma cell lines from the Cancer Cell Line Encyclopedia project. *J. Virol.* 89 713–729. 10.1128/jvi.02570-14 25355872 PMC4301145

[B21] Cesarman E. Chang Y. Moore P. S. Said J. W. Knowles D. M. (1995). Kaposi’s sarcoma-associated herpesvirus-like DNA sequences in AIDS-related body-cavity-based lymphomas. *N. Engl. J. Med.* 332 1186–1191. 10.1056/nejm199505043321802 7700311

[B22] Cesarman E. Nador R. G. Aozasa K. Delsol G. Said J. W. Knowles D. M. (1996). Kaposi’s sarcoma-associated herpesvirus in non-AIDS related lymphomas occurring in body cavities. *Am. J. Pathol.* 149 53–57. 8686762 PMC1865234

[B23] Chamseddin B. H. Lee E. E. Kim J. Zhan X. Yang R. Murphy K. M. (2019). Assessment of circularized E7 RNA, GLUT1, and PD-L1 in anal squamous cell carcinoma. *Oncotarget* 10 5958–5969. 10.18632/oncotarget.27234 31666927 PMC6800260

[B24] Chan K. C. A. Woo J. K. S. King A. Zee B. C. Y. Lam W. K. J. Chan S. L. (2017). Analysis of Plasma Epstein-Barr virus DNA to screen for nasopharyngeal cancer. *N. Engl. J. Med.* 377 513–522. 28792880 10.1056/NEJMoa1701717

[B25] Chandriani S. Xu Y. Ganem D. (2010). The lytic transcriptome of Kaposi’s sarcoma-associated herpesvirus reveals extensive transcription of noncoding regions, including regions antisense to important genes. *J. Virol.* 84 7934–7942. 10.1128/jvi.00645-10 20534856 PMC2916530

[B26] Chang Y. Cesarman E. Pessin M. S. Lee F. Culpepper J. Knowles D. M. (1994). Identification of herpesvirus-like DNA sequences in AIDS-associated Kaposi’s sarcoma. *Science* 266 1865–1869. 10.1126/science.7997879 7997879

[B27] Chen J. Li Y. Zheng Q. Bao C. He J. Chen B. (2017). Circular RNA profile identifies circPVT1 as a proliferative factor and prognostic marker in gastric cancer. *Cancer Lett.* 388 208–219. 10.1016/j.canlet.2016.12.006 27986464

[B28] Chen S. Li T. Zhao Q. Xiao B. Guo J. (2017). Using circular RNA hsa_circ_0000190 as a new biomarker in the diagnosis of gastric cancer. *Clin. Chim. Acta* 466 167–171. 10.1016/j.cca.2017.01.025 28130019

[B29] Chen Y. G. Kim M. V. Chen X. Batista P. J. Aoyama S. Wilusz J. E. (2017). Sensing self and foreign circular RNAs by intron identity. *Mol. Cell.* 67 228.e225–238.e225. 28625551 10.1016/j.molcel.2017.05.022 PMC5610545

[B30] Chen L. L. Yang L. (2015). Regulation of circRNA biogenesis. *RNA Biol.* 12 381–388. 10.1080/15476286.2015.1020271 25746834 PMC4615371

[B31] Chen Y. G. Chen R. Ahmad S. Verma R. Kasturi S. P. Amaya L. (2019). N6-methyladenosine modification controls circular RNA immunity. *Mol. Cell.* 76 96.e109–109.e109. 31474572 10.1016/j.molcel.2019.07.016 PMC6778039

[B32] Chiang A. K. Tao Q. Srivastava G. Ho F. C. (1996). Nasal NK- and T-cell lymphomas share the same type of Epstein-Barr virus latency as nasopharyngeal carcinoma and Hodgkin’s disease. *Int. J. Cancer* 68 285–290. 10.1002/(sici)1097-0215(19961104)68:3<285::aid-ijc3>3.0.co;2-y 8903467

[B33] Cocquerelle C. Mascrez B. Hetuin D. Bailleul B. (1993). Mis-splicing yields circular RNA molecules. *FASEB J.* 7 155–160. 10.1096/fasebj.7.1.7678559 7678559

[B34] Concha M. Wang X. Cao S. Baddoo M. Fewell C. Lin Z. (2012). Identification of new viral genes and transcript isoforms during Epstein-Barr virus reactivation using RNA-Seq. *J. Virol.* 86 1458–1467. 10.1128/jvi.06537-11 22090128 PMC3264377

[B35] Conn S. J. Pillman K. A. Toubia J. Conn V. M. Salmanidis M. Phillips C. A. (2015). The RNA binding protein quaking regulates formation of circRNAs. *Cell* 160 1125–1134. 10.1016/j.cell.2015.02.014 25768908

[B36] Danan M. Schwartz S. Edelheit S. Sorek R. (2012). Transcriptome-wide discovery of circular RNAs in Archaea. *Nucleic Acids Res.* 40 3131–3142. 10.1093/nar/gkr1009 22140119 PMC3326292

[B37] de Sanjose S. Bosch R. Schouten T. Verkuijlen S. Nieters A. Foretova L. (2007). Epstein-Barr virus infection and risk of lymphoma: immunoblot analysis of antibody responses against EBV-related proteins in a large series of lymphoma subjects and matched controls. *Int. J. Cancer* 121 1806–1812. 10.1002/ijc.22857 17557295

[B38] Deacon E. M. Pallesen G. Niedobitek G. Crocker J. Brooks L. Rickinson A. B. (1993). Epstein-Barr virus and Hodgkin’s disease: transcriptional analysis of virus latency in the malignant cells. *J. Exp. Med.* 177 339–349. 10.1084/jem.177.2.339 8381153 PMC2190903

[B39] Dresang L. R. Teuton J. R. Feng H. Jacobs J. M. Camp D. G. II Purvine S. O. (2011). Coupled transcriptome and proteome analysis of human lymphotropic tumor viruses: insights on the detection and discovery of viral genes. *BMC Genomics* 12:625. 10.1186/1471-2164-12-625 22185355 PMC3282826

[B40] Du W. W. Yang W. Liu E. Yang Z. Dhaliwal P. Yang B. B. (2016). Foxo3 circular RNA retards cell cycle progression via forming ternary complexes with p21 and CDK2. *Nucleic Acids Res.* 44 2846–2858. 10.1093/nar/gkw027 26861625 PMC4824104

[B41] Edwards R. H. Marquitz A. R. Raab-Traub N. (2008). Epstein-Barr virus BART microRNAs are produced from a large intron prior to splicing. *J. Virol.* 82 9094–9106. 10.1128/jvi.00785-08 18614630 PMC2546912

[B42] Errichelli L. Dini Modigliani S. Laneve P. Colantoni A. Legnini I. Capauto D. (2017). FUS affects circular RNA expression in murine embryonic stem cell-derived motor neurons. *Nat. Commun.* 8:14741. 10.1038/ncomms14741 28358055 PMC5379105

[B43] Fan X. Zhang X. Wu X. Guo H. Hu Y. Tang F. (2015). Single-cell RNA-seq transcriptome analysis of linear and circular RNAs in mouse preimplantation embryos. *Genome Biol.* 16:148. 10.1186/s13059-015-0706-1 PMC4511241 26201400

[B44] Feng H. Shuda M. Chang Y. Moore P. S. (2008). Clonal integration of a polyomavirus in human Merkel cell carcinoma. *Science* 319 1096–1100. 10.1126/science.1152586 18202256 PMC2740911

[B45] Gong L. P. Chen J. N. Dong M. Xiao Z. D. Feng Z. Y. Pan Y. H. (2020). Epstein-Barr virus-derived circular RNA LMP2A induces stemness in EBV-associated gastric cancer. *EMBO Rep.* 21:e49689. 10.15252/embr.201949689 PMC7534631 32790025

[B46] Gottwein E. Corcoran D. L. Mukherjee N. Skalsky R. L. Hafner M. Nusbaum J. D. (2011). Viral microRNA targetome of KSHV-infected primary effusion lymphoma cell lines. *Cell Host Microbe* 10 515–526. 10.1016/j.chom.2011.09.012 22100165 PMC3222872

[B47] Guo J. N. Li J. Zhu C. L. Feng W. T. Shao J. X. Wan L. (2016). Comprehensive profile of differentially expressed circular RNAs reveals that hsa_circ_0000069 is upregulated and promotes cell proliferation, migration, and invasion in colorectal cancer. *Onco Targets Ther.* 9 7451–7458. 10.2147/ott.s123220 28003761 PMC5158168

[B48] Guo J. U. Agarwal V. Guo H. Bartel D. P. (2014). Expanded identification and characterization of mammalian circular RNAs. *Genome Biol.* 15:409. 10.1186/s13059-014-0409-z PMC4165365 25070500

[B49] Hansen A. Henderson S. Lagos D. Nikitenko L. Coulter E. Roberts S. (2010). KSHV-encoded miRNAs target MAF to induce endothelial cell reprogramming. *Genes Dev.* 24 195–205. 10.1101/gad.553410 20080955 PMC2807354

[B50] Hansen T. B. Jensen T. I. Clausen B. H. Bramsen J. B. Finsen B. Damgaard C. K. (2013). Natural RNA circles function as efficient microRNA sponges. *Nature* 495 384–388. 10.1038/nature11993 23446346

[B51] Hiriart E. Gruffat H. Buisson M. Mikaelian I. Keppler S. Meresse P. (2005). Interaction of the Epstein-Barr virus mRNA export factor EB2 with human Spen proteins SHARP, OTT1, and a novel member of the family, OTT3, links Spen proteins with splicing regulation and mRNA export. *J. Biol. Chem.* 280 36935–36945. 10.1074/jbc.m501725200 16129689

[B52] Hitt M. M. Allday M. J. Hara T. Karran L. Jones M. D. Busson P. (1989). EBV gene expression in an NPC-related tumour. *EMBO J.* 8 2639–2651. 10.1002/j.1460-2075.1989.tb08404.x 2479554 PMC401270

[B53] Holdt L. M. Stahringer A. Sass K. Pichler G. Kulak N. A. Wilfert W. (2016). Circular non-coding RNA ANRIL modulates ribosomal RNA maturation and atherosclerosis in humans. *Nat. Commun.* 7:12429. 10.1038/ncomms12429 PMC4992165 27539542

[B54] Hosseinipour M. C. Sweet K. M. Xiong J. Namarika D. Mwafongo A. Nyirenda M. (2014). Viral profiling identifies multiple subtypes of Kaposi’s sarcoma. *mBio* 5:e1633-14. 10.1128/mBio.01633-14 PMC4173763 25249280

[B55] Hsiao K. Y. Lin Y. C. Gupta S. K. Chang N. Yen L. Sun H. S. (2017). Noncoding effects of circular RNA CCDC66 promote colon cancer growth and metastasis. *Cancer Res.* 77 2339–2350. 10.1158/0008-5472.can-16-1883 28249903 PMC5910173

[B56] Hsu M. T. Coca-Prados M. (1979). Electron microscopic evidence for the circular form of RNA in the cytoplasm of eukaryotic cells. *Nature* 280 339–340. 10.1038/280339a0 460409

[B57] Huang G. Zhu H. Shi Y. Wu W. Cai H. Chen X. (2015). cir-ITCH plays an inhibitory role in colorectal cancer by regulating the Wnt/beta-catenin pathway. *PLoS One* 10:e0131225. 10.1371/journal.pone.0131225 26110611 PMC4482251

[B58] Huang J. T. Chen J. N. Gong L. P. Bi Y. H. Liang J. Zhou L. (2019). Identification of virus-encoded circular RNA. *Virology* 529 144–151. 10.1016/j.virol.2019.01.014 30710798

[B59] Huang S. Yang B. Chen B. J. Bliim N. Ueberham U. Arendt T. (2017). The emerging role of circular RNAs in transcriptome regulation. *Genomics* 109 401–407. 10.1016/j.ygeno.2017.06.005 28655641

[B60] Hutzinger R. Feederle R. Mrazek J. Schiefermeier N. Balwierz P. J. Zavolan M. (2009). Expression and processing of a small nucleolar RNA from the Epstein-Barr virus genome. *PLoS Pathog.* 5:e1000547. 10.1371/journal.ppat.1000547 19680535 PMC2718842

[B61] Ivanov A. Memczak S. Wyler E. Torti F. Porath H. T. Orejuela M. R. (2015). Analysis of intron sequences reveals hallmarks of circular RNA biogenesis in animals. *Cell Rep.* 10 170–177. 10.1016/j.celrep.2014.12.019 25558066

[B62] Jacobs S. R. Damania B. (2011). The viral interferon regulatory factors of KSHV: immunosuppressors or oncogenes? *Front. Immunol.* 2:19. 10.3389/fimmu.2011.00019 22566809 PMC3342017

[B63] Jeck W. R. Sharpless N. E. (2014). Detecting and characterizing circular RNAs. *Nat. Biotechnol.* 32 453–461. 10.1038/nbt.2890 24811520 PMC4121655

[B64] Jeck W. R. Sorrentino J. A. Wang K. Slevin M. K. Burd C. E. Liu J. (2013). Circular RNAs are abundant, conserved, and associated with ALU repeats. *RNA* 19 141–157. 10.1261/rna.035667.112 23249747 PMC3543092

[B65] Juillard F. Bazot Q. Mure F. Tafforeau L. Macri C. Rabourdin-Combe C. (2012). Epstein-Barr virus protein EB2 stimulates cytoplasmic mRNA accumulation by counteracting the deleterious effects of SRp20 on viral mRNAs. *Nucleic Acids Res.* 40 6834–6849. 10.1093/nar/gks319 22505578 PMC3413128

[B66] Juillard F. Hiriart E. Sergeant N. Vingtdeux-Didier V. Drobecq H. Sergeant A. (2009). Epstein-Barr virus protein EB2 contains an N-terminal transferable nuclear export signal that promotes nucleocytoplasmic export by directly binding TAP/NXF1. *J. Virol.* 83 12759–12768. 10.1128/jvi.01276-09 19793817 PMC2786861

[B67] Katano H. Sato Y. Kurata T. Mori S. Sata T. (2000). Expression and localization of human herpesvirus 8-encoded proteins in primary effusion lymphoma, Kaposi’s sarcoma, and multicentric Castleman’s disease. *Virology* 269 335–344. 10.1006/viro.2000.0196 10753712

[B68] Kheir F. Zhao M. Strong M. J. Yu Y. Nanbo A. Flemington E. K. (2019). Detection of epstein-barr virus infection in non-small cell lung cancer. *Cancers* 11:759. 10.3390/cancers11060759 31159203 PMC6627930

[B69] Kleaveland B. Shi C. Y. Stefano J. Bartel D. P. (2018). A network of noncoding regulatory RNAs acts in the mammalian brain. *Cell* 174 350.e317–362.e317. 29887379 10.1016/j.cell.2018.05.022 PMC6559361

[B70] Kolakofsky D. (1976). Isolation and characterization of sendai virus DI-RNAs. *Cell* 8 547–555. 10.1016/0092-8674(76)90223-3 182384

[B71] Kramer M. C. Liang D. Tatomer D. C. Gold B. March Z. M. Cherry S. (2015). Combinatorial control of *Drosophila* circular RNA expression by intronic repeats, hnRNPs, and SR proteins. *Genes Dev.* 29 2168–2182. 10.1101/gad.270421.115 26450910 PMC4617980

[B72] Kristensen L. S. Okholm T. L. H. Veno M. T. Kjems J. (2018). Circular RNAs are abundantly expressed and upregulated during human epidermal stem cell differentiation. *RNA Biol.* 15 280–291. 10.1080/15476286.2017.1409931 29283313 PMC5798954

[B73] Laichalk L. L. Thorley-Lawson D. A. (2005). Terminal differentiation into plasma cells initiates the replicative cycle of Epstein-Barr virus in vivo. *J. Virol.* 79 1296–1307. 10.1128/jvi.79.2.1296-1307.2005 15613356 PMC538585

[B74] Lee H. R. Kim M. H. Lee J. S. Liang C. Jung J. U. (2009). Viral interferon regulatory factors. *J. Interferon Cytokine Res.* 29 621–627. 10.1089/jir.2009.0067 19715458 PMC2956608

[B75] Legnini I. Di Timoteo G. Rossi F. Morlando M. Briganti F. Sthandier O. (2017). Circ-ZNF609 is a circular RNA that can be translated and functions in myogenesis. *Mol. Cell.* 66 22.e29–37.e29. 28344082 10.1016/j.molcel.2017.02.017 PMC5387670

[B76] Lei X. Bai Z. Ye F. Huang Y. Gao S. J. (2010a). Regulation of herpesvirus lifecycle by viral microRNAs. *Virulence* 1 433–435. 10.4161/viru.1.5.12966 21170300 PMC3003333

[B77] Lei X. Bai Z. Ye F. Xie J. Kim C. G. Huang Y. (2010b). Regulation of NF-kappaB inhibitor IkappaBalpha and viral replication by a KSHV microRNA. *Nat. Cell Biol.* 12 193–199. 10.1038/ncb2019 20081837 PMC2815189

[B78] Leung S. F. Zee B. Ma B. B. Hui E. P. Mo F. Lai M. (2006). Plasma Epstein-Barr viral deoxyribonucleic acid quantitation complements tumor-node-metastasis staging prognostication in nasopharyngeal carcinoma. *J. Clin. Oncol.* 24 5414–5418. 10.1200/jco.2006.07.7982 17135642

[B79] Li H. Hao X. Wang H. Liu Z. He Y. Pu M. (2016). Circular RNA expression profile of pancreatic ductal adenocarcinoma revealed by microarray. *Cell Physiol. Biochem.* 40 1334–1344. 10.1159/000453186 27997903

[B80] Li P. Chen H. Chen S. Mo X. Li T. Xiao B. (2017). Circular RNA 0000096 affects cell growth and migration in gastric cancer. *Br. J. Cancer* 116 626–633. 10.1038/bjc.2016.451 28081541 PMC5344286

[B81] Li W. H. Song Y. C. Zhang H. Zhou Z. J. Xie X. Zeng Q. N. (2017). Decreased expression of Hsa_circ_00001649 in gastric cancer and its clinical significance. *Dis. Markers* 2017:4587698. 10.1155/2017/4587698 PMC5266807 28167847

[B82] Li P. Chen S. Chen H. Mo X. Li T. Shao Y. (2015). Using circular RNA as a novel type of biomarker in the screening of gastric cancer. *Clin. Chim. Acta* 444 132–136. 10.1016/j.cca.2015.02.018 25689795

[B83] Li Y. Zheng Q. Bao C. Li S. Guo W. Zhao J. (2015). Circular RNA is enriched and stable in exosomes: a promising biomarker for cancer diagnosis. *Cell Res.* 25 981–984. 10.1038/cr.2015.82 26138677 PMC4528056

[B84] Li Z. Huang C. Bao C. Chen L. Lin M. Wang X. (2015). Exon-intron circular RNAs regulate transcription in the nucleus. *Nat. Struct. Mol. Biol.* 22 256–264. 10.1038/nsmb.2959 25664725

[B85] Liang D. Tatomer D. C. Luo Z. Wu H. Yang L. Chen L. L. (2017). The output of protein-coding genes shifts to circular RNAs when the Pre-mRNA processing machinery is limiting. *Mol. Cell.* 68 940.e943–954.e943. 29174924 10.1016/j.molcel.2017.10.034 PMC5728686

[B86] Lin Z. Wang X. Strong M. J. Concha M. Baddoo M. Xu G. (2013). Whole-genome sequencing of the Akata and Mutu Epstein-Barr virus strains. *J. Virol.* 87 1172–1182. 10.1128/jvi.02517-12 23152513 PMC3554088

[B87] Lin Z. Xu G. Deng N. Taylor C. Zhu D. Flemington E. K. (2010). Quantitative and qualitative RNA-Seq-based evaluation of Epstein-Barr virus transcription in type I latency Burkitt’s lymphoma cells. *J. Virol.* 84 13053–13058. 10.1128/jvi.01521-10 20943983 PMC3004295

[B88] Liu Q. Shuai M. Xia Y. (2019). Knockdown of EBV-encoded circRNA circRPMS1 suppresses nasopharyngeal carcinoma cell proliferation and metastasis through sponging multiple miRNAs. *Cancer Manag. Res.* 11 8023–8031. 10.2147/cmar.s218967 31695488 PMC6717849

[B89] Liu Z. Zhou Y. Liang G. Ling Y. Tan W. Tan L. (2019). Circular RNA hsa_circ_001783 regulates breast cancer progression via sponging miR-200c-3p. *Cell Death Dis.* 10:55. 10.1038/s41419-018-1287-1 PMC6343010 30670688

[B90] Lo Y. M. Chan L. Y. Lo K. W. Leung S. F. Zhang J. Chan A. T. (1999). Quantitative analysis of cell-free Epstein-Barr virus DNA in plasma of patients with nasopharyngeal carcinoma. *Cancer Res.* 59 1188–1191. 10096545

[B91] Longnecker R. Kieff E. (1990). A second Epstein-Barr virus membrane protein (LMP2) is expressed in latent infection and colocalizes with LMP1. *J. Virol.* 64 2319–2326. 10.1128/jvi.64.5.2319-2326.1990 2157888 PMC249393

[B92] Lu C. C. Jeng Y. Y. Tsai C. H. Liu M. Y. Yeh S. W. Hsu T. Y. (2006). Genome-wide transcription program and expression of the Rta responsive gene of Epstein-Barr virus. *Virology* 345 358–372. 10.1016/j.virol.2005.09.064 16298410

[B93] Lu M. Zhang Z. Xue M. Zhao B. S. Harder O. Li A. (2020). N(6)-methyladenosine modification enables viral RNA to escape recognition by RNA sensor RIG-I. *Nat. Microbiol.* 5 584–598. 10.1038/s41564-019-0653-9 32015498 PMC7137398

[B94] Majerciak V. Ni T. Yang W. Meng B. Zhu J. Zheng Z. M. (2013). A viral genome landscape of RNA polyadenylation from KSHV latent to lytic infection. *PLoS Pathog.* 9:e1003749. 10.1371/journal.ppat.1003749 24244170 PMC3828183

[B95] Memczak S. Jens M. Elefsinioti A. Torti F. Krueger J. Rybak A. (2013). Circular RNAs are a large class of animal RNAs with regulatory potency. *Nature* 495 333–338. 10.1038/nature11928 23446348

[B96] Mesri E. A. Feitelson M. A. Munger K. (2014). Human viral oncogenesis: a cancer hallmarks analysis. *Cell Host Microbe* 15 266–282. 10.1016/j.chom.2014.02.011 24629334 PMC3992243

[B97] Moore P. S. Chang Y. (2017). Common commensal cancer viruses. *PLoS Pathog.* 13:e1006078. 10.1371/journal.ppat.1006078 28103305 PMC5245784

[B98] Moss W. N. Steitz J. A. (2013). Genome-wide analyses of Epstein-Barr virus reveal conserved RNA structures and a novel stable intronic sequence RNA. *BMC Genomics* 14:543. 10.1186/1471-2164-14-543 23937650 PMC3751371

[B99] Mure F. Panthu B. Zanella-Cleon I. Delolme F. Manet E. Ohlmann T. (2018). Epstein-Barr virus protein EB2 stimulates translation initiation of mRNAs through direct interactions with both Poly(A)-binding protein and eukaryotic initiation factor 4G. *J. Virol.* 92:e1917-17. 10.1128/JVI.01917-17 PMC5774873 29142127

[B100] Noto J. J. Schmidt C. A. Matera A. G. (2017). Engineering and expressing circular RNAs via tRNA splicing. *RNA Biol.* 14 978–984. 10.1080/15476286.2017.1317911 28402213 PMC5680671

[B101] O’Grady T. Cao S. Strong M. J. Concha M. Wang X. Splinter Bondurant S. (2014). Global bidirectional transcription of the Epstein-Barr virus genome during reactivation. *J. Virol.* 88 1604–1616. 10.1128/jvi.02989-13 24257595 PMC3911580

[B102] O’Grady T. Wang X. Honer Zu Bentrup K. Baddoo M. Concha M. Flemington E. K. (2016). Global transcript structure resolution of high gene density genomes through multi-platform data integration. *Nucleic Acids Res.* 44:e145. 10.1093/nar/gkw629 27407110 PMC5062983

[B103] Pamudurti N. R. Bartok O. Jens M. Ashwal-Fluss R. Stottmeister C. Ruhe L. (2017). Translation of CircRNAs. *Mol. Cell.* 66 9–21.e7. 28344080 10.1016/j.molcel.2017.02.021 PMC5387669

[B104] Pamudurti N. R. Konakondla-Jacob V. V. Krishnamoorthy A. Ashwal-Fluss R. Bartok O. Wüst S. (2018). An in vivo knockdown strategy reveals multiple functions for circMbl. *bioRxiv [Preprint]* 10.1101/483271

[B105] Parravicini C. Chandran B. Corbellino M. Berti E. Paulli M. Moore P. S. (2000). Differential viral protein expression in Kaposi’s sarcoma-associated herpesvirus-infected diseases: Kaposi’s sarcoma, primary effusion lymphoma, and multicentric Castleman’s disease. *Am. J. Pathol.* 156 743–749. 10702388 10.1016/S0002-9440(10)64940-1 PMC1876837

[B106] Pfeffer S. Sewer A. Lagos-Quintana M. Sheridan R. Sander C. Grasser F. A. (2005). Identification of microRNAs of the herpesvirus family. *Nat. Methods* 2 269–276. 15782219 10.1038/nmeth746

[B107] Pfeffer S. Zavolan M. Grasser F. A. Chien M. Russo J. J. Ju J. (2004). Identification of virus-encoded microRNAs. *Science* 304 734–736. 10.1126/science.1096781 15118162

[B108] Piwecka M. Glazar P. Hernandez-Miranda L. R. Memczak S. Wolf S. A. Rybak-Wolf A. (2017). Loss of a mammalian circular RNA locus causes miRNA deregulation and affects brain function. *Science* 357:eaam8526. 10.1126/science.aam8526 28798046

[B109] Qiao Y. Zhao X. Liu J. Yang W. (2019). Epstein-Barr virus circRNAome as host miRNA sponge regulates virus infection, cell cycle, and oncogenesis. *Bioengineered* 10 593–603. 10.1080/21655979.2019.1679698 31668120 PMC6844377

[B110] Qin M. Liu G. Huo X. Tao X. Sun X. Ge Z. (2016). Hsa_circ_0001649: a circular RNA and potential novel biomarker for hepatocellular carcinoma. *Cancer Biomark.* 16 161–169. 26600397 10.3233/CBM-150552 PMC13016540

[B111] Qin Z. Freitas E. Sullivan R. Mohan S. Bacelieri R. Branch D. (2010). Upregulation of xCT by KSHV-encoded microRNAs facilitates KSHV dissemination and persistence in an environment of oxidative stress. *PLoS Pathog.* 6:e1000742. 10.1371/journal.ppat.1000742 20126446 PMC2813276

[B112] Raab-Traub N. Dambaugh T. Kieff E. (1980). DNA of Epstein-Barr virus VIII: B95-8, the previous prototype, is an unusual deletion derivative. *Cell* 22 257–267. 10.1016/0092-8674(80)90173-7 6253079

[B113] Rennekamp A. J. Lieberman P. M. (2011). Initiation of Epstein-Barr virus lytic replication requires transcription and the formation of a stable RNA-DNA hybrid molecule at OriLyt. *J. Virol.* 85 2837–2850. 10.1128/jvi.02175-10 21191028 PMC3067963

[B114] Rickinson A. Kieff E. (2007). “Epstein-Barr virus,” in *Fields Virology*, 5th Edn, eds Knipe D. Howley P. (Philadelphia, PA: Lippincott Williams & Wilkins), 2655–2700.

[B115] Roden R. B. S. Stern P. L. (2018). Opportunities and challenges for human papillomavirus vaccination in cancer. *Nat. Rev. Cancer* 18 240–254. 10.1038/nrc.2018.13 29497146 PMC6454884

[B116] Rossetto C. C. Pari G. (2012). KSHV PAN RNA associates with demethylases UTX and JMJD3 to activate lytic replication through a physical interaction with the virus genome. *PLoS Pathog.* 8:e1002680. 10.1371/journal.ppat.1002680 22589717 PMC3349751

[B117] Rossetto C. C. Pari G. S. (2011). Kaposi’s sarcoma-associated herpesvirus noncoding polyadenylated nuclear RNA interacts with virus- and host cell-encoded proteins and suppresses expression of genes involved in immune modulation. *J. Virol.* 85 13290–13297. 10.1128/jvi.05886-11 21957289 PMC3233155

[B118] Rossetto C. C. Pari G. S. (2014). PAN’s labyrinth: molecular biology of Kaposi’s sarcoma-associated herpesvirus (KSHV) PAN RNA, a multifunctional long noncoding RNA. *Viruses* 6 4212–4226. 10.3390/v6114212 25375885 PMC4246217

[B119] Rossetto C. C. Tarrant-Elorza M. Verma S. Purushothaman P. Pari G. S. (2013). Regulation of viral and cellular gene expression by Kaposi’s sarcoma-associated herpesvirus polyadenylated nuclear RNA. *J. Virol.* 87 5540–5553. 10.1128/jvi.03111-12 23468496 PMC3648157

[B120] Rovedo M. Longnecker R. (2007). Epstein-barr virus latent membrane protein 2B (LMP2B) modulates LMP2A activity. *J. Virol.* 81 84–94. 10.1128/jvi.01302-06 17035319 PMC1797235

[B121] Rybak-Wolf A. Stottmeister C. Glazar P. Jens M. Pino N. Giusti S. (2015). Circular RNAs in the mammalian brain are highly abundant, conserved, and dynamically expressed. *Mol. Cell* 58 870–885. 10.1016/j.molcel.2015.03.027 25921068

[B122] Salzman J. Chen R. E. Olsen M. N. Wang P. L. Brown P. O. (2013). Cell-type specific features of circular RNA expression. *PLoS Genet.* 9:e1003777. 10.1371/journal.pgen.1003777 24039610 PMC3764148

[B123] Salzman J. Gawad C. Wang P. L. Lacayo N. Brown P. O. (2012). Circular RNAs are the predominant transcript isoform from hundreds of human genes in diverse cell types. *PLoS One* 7:e30733. 10.1371/journal.pone.0030733 22319583 PMC3270023

[B124] Samols M. A. Hu J. Skalsky R. L. Renne R. (2005). Cloning and identification of a microRNA cluster within the latency-associated region of Kaposi’s sarcoma-associated herpesvirus. *J. Virol.* 79 9301–9305. 10.1128/jvi.79.14.9301-9305.2005 15994824 PMC1168752

[B125] Sand M. Bechara F. G. Gambichler T. Sand D. Bromba M. Hahn S. A. (2016a). Circular RNA expression in cutaneous squamous cell carcinoma. *J. Dermatol. Sci.* 83 210–218. 27298156 10.1016/j.jdermsci.2016.05.012

[B126] Sand M. Bechara F. G. Sand D. Gambichler T. Hahn S. A. Bromba M. (2016b). Circular RNA expression in basal cell carcinoma. *Epigenomics* 8 619–632. 10.2217/epi-2015-0019 27097056

[B127] Sanger H. L. Klotz G. Riesner D. Gross H. J. Kleinschmidt A. K. (1976). Viroids are single-stranded covalently closed circular RNA molecules existing as highly base-paired rod-like structures. *Proc. Natl. Acad. Sci. U.S.A.* 73 3852–3856. 10.1073/pnas.73.11.3852 1069269 PMC431239

[B128] Sarid R. Flore O. Bohenzky R. A. Chang Y. Moore P. S. (1998). Transcription mapping of the Kaposi’s sarcoma-associated herpesvirus (human herpesvirus 8) genome in a body cavity-based lymphoma cell line (BC-1). *J. Virol.* 72 1005–1012. 10.1128/jvi.72.2.1005-1012.1998 9444993 PMC124571

[B129] Sathish N. Wang X. Yuan Y. (2012). Tegument proteins of Kaposi’s sarcoma-associated herpesvirus and related gamma-herpesviruses. *Front. Microbiol.* 3:98. 10.3389/fmicb.2012.00098 22435068 PMC3304090

[B130] Schifano J. M. Corcoran K. Kelkar H. Dittmer D. P. (2017). Expression of the antisense-to-latency transcript long noncoding RNA in Kaposi’s sarcoma-associated herpesvirus. *J. Virol.* 91:e1698-16. 10.1128/JVI.01698-16 PMC5286886 27928018

[B131] Sekiba K. Otsuka M. Ohno M. Kishikawa T. Yamagami M. Suzuki T. (2018). DHX9 regulates production of hepatitis B virus-derived circular RNA and viral protein levels. *Oncotarget* 9 20953–20964. 10.18632/oncotarget.25104 29765512 PMC5940377

[B132] Semmes O. J. Chen L. Sarisky R. T. Gao Z. Zhong L. Hayward S. D. (1998). Mta has properties of an RNA export protein and increases cytoplasmic accumulation of Epstein-Barr virus replication gene mRNA. *J. Virol.* 72 9526–9534. 10.1128/jvi.72.12.9526-9534.1998 9811685 PMC110453

[B133] Seo G. J. Chen C. J. Sullivan C. S. (2009). Merkel cell polyomavirus encodes a microRNA with the ability to autoregulate viral gene expression. *Virology* 383 183–187. 10.1016/j.virol.2008.11.001 19046593

[B134] Sergeant A. Gruffat H. Manet E. (2008). The Epstein-Barr virus (EBV) protein EB is an mRNA export factor essential for virus production. *Front. Biosci.* 13:3798–3813. 10.2741/2969 18508475

[B135] Shang X. Li G. Liu H. Li T. Liu J. Zhao Q. (2016). Comprehensive circular RNA profiling reveals that hsa_circ_0005075, a new circular RNA biomarker. is involved in hepatocellular crcinoma development. *Medicine* 95:e3811. 10.1097/md.0000000000003811 27258521 PMC4900729

[B136] Shannon-Lowe C. Rickinson A. (2019). The global landscape of EBV-associated tumors. *Front. Oncol.* 9:713. 10.3389/fonc.2019.00713 31448229 PMC6691157

[B137] Shuda M. Feng H. Kwun H. J. Rosen S. T. Gjoerup O. Moore P. S. (2008). T antigen mutations are a human tumor-specific signature for Merkel cell polyomavirus. *Proc. Natl. Acad. Sci. U.S.A.* 105 16272–16277. 10.1073/pnas.0806526105 18812503 PMC2551627

[B138] Soulier J. Grollet L. Oksenhendler E. Cacoub P. Cazals-Hatem D. Babinet P. (1995). Kaposi’s sarcoma-associated herpesvirus-like DNA sequences in multicentric Castleman’s disease. *Blood* 86 1276–1280. 7632932

[B139] Staskus K. A. Zhong W. Gebhard K. Herndier B. Wang H. Renne R. (1997). Kaposi’s sarcoma-associated herpesvirus gene expression in endothelial (spindle) tumor cells. *J. Virol.* 71 715–719. 10.1128/jvi.71.1.715-719.1997 8985403 PMC191104

[B140] Strong M. J. O’grady T. Lin Z. Xu G. Baddoo M. Parsons C. (2013a). Epstein-Barr virus and human herpesvirus 6 detection in a non-Hodgkin’s diffuse large B-cell lymphoma cohort by using RNA sequencing. *J. Virol.* 87 13059–13062. 10.1128/jvi.02380-13 24049168 PMC3838131

[B141] Strong M. J. Xu G. Coco J. Baribault C. Vinay D. S. Lacey M. R. (2013b). Differences in gastric carcinoma microenvironment stratify according to EBV infection intensity: implications for possible immune adjuvant therapy. *PLoS Pathog.* 9:e1003341. 10.1371/journal.ppat.1003341 23671415 PMC3649992

[B142] Sugiura M. Imai S. Tokunaga M. Koizumi S. Uchizawa M. Okamoto K. (1996). Transcriptional analysis of Epstein-Barr virus gene expression in EBV-positive gastric carcinoma: unique viral latency in the tumour cells. *Br. J. Cancer* 74 625–631. 10.1038/bjc.1996.412 8761381 PMC2074674

[B143] Sui W. Shi Z. Xue W. Ou M. Zhu Y. Chen J. (2017). Circular RNA and gene expression profiles in gastric cancer based on microarray chip technology. *Oncol. Rep.* 37 1804–1814. 10.3892/or.2017.5415 28184940

[B144] Sun C. C. Thorley-Lawson D. A. (2007). Plasma cell-specific transcription factor XBP-1s binds to and transactivates the Epstein-Barr virus BZLF1 promoter. *J. Virol.* 81 13566–13577. 10.1128/jvi.01055-07 17898050 PMC2168822

[B145] Tagawa T. Gao S. Koparde V. N. Gonzalez M. Spouge J. L. Serquina A. P. (2018). Discovery of Kaposi’s sarcoma herpesvirus-encoded circular RNAs and a human antiviral circular RNA. *Proc. Natl. Acad. Sci. U.S.A.* 115 12805–12810. 10.1073/pnas.1816183115 30455306 PMC6294913

[B146] Tao Q. Robertson K. D. Manns A. Hildesheim A. Ambinder R. F. (1998). Epstein-Barr virus (EBV) in endemic Burkitt’s lymphoma: molecular analysis of primary tumor tissue. *Blood* 91 1373–1381. 10.1182/blood.v91.4.1373 9454768

[B147] Taylor J. L. Bennett H. N. Snyder B. A. Moore P. S. Chang Y. (2005). Transcriptional analysis of latent and inducible Kaposi’s sarcoma-associated herpesvirus transcripts in the K4 to K7 region. *J. Virol.* 79 15099–15106. 10.1128/jvi.79.24.15099-15106.2005 16306581 PMC1315995

[B148] Theiss J. M. Gunther T. Alawi M. Neumann F. Tessmer U. Fischer N. (2015). A comprehensive analysis of replicating merkel cell polyomavirus genomes delineates the viral transcription program and suggests a role for mcv-miR-M1 in episomal persistence. *PLoS Pathog.* 11:e1004974. 10.1371/journal.ppat.1004974 26218535 PMC4517807

[B149] Tolstov Y. L. Pastrana D. V. Feng H. Becker J. C. Jenkins F. J. Moschos S. (2009). Human Merkel cell polyomavirus infection II. MCV is a common human infection that can be detected by conformational capsid epitope immunoassays. *Int. J. Cancer* 125 1250–1256. 10.1002/ijc.24509 19499548 PMC2747737

[B150] Toptan T. Abere B. Nalesnik M. A. Swerdlow S. H. Ranganathan S. Lee N. (2018). Circular DNA tumor viruses make circular RNAs. *Proc. Natl. Acad. Sci. U.S.A.* 115 E8737–E8745. 30150410 10.1073/pnas.1811728115 PMC6140489

[B151] Torresi J. Tran B. M. Christiansen D. Earnest-Silveira L. Schwab R. H. M. Vincan E. (2019). HBV-related hepatocarcinogenesis: the role of signalling pathways and innovative ex vivo research models. *BMC Cancer* 19:707. 10.1186/s12885-019-5916-6 31319796 PMC6637598

[B152] Umbach J. L. Cullen B. R. (2010). In-depth analysis of Kaposi’s sarcoma-associated herpesvirus microRNA expression provides insights into the mammalian microRNA-processing machinery. *J. Virol.* 84 695–703. 10.1128/jvi.02013-09 19889781 PMC2798371

[B153] Ungerleider N. Concha M. Lin Z. Roberts C. Wang X. Cao S. (2018). The Epstein Barr virus circRNAome. *PLoS Pathog.* 14:e1007206. 10.1371/journal.ppat.1007206 30080890 PMC6095625

[B154] Ungerleider N. A. Jain V. Wang Y. Maness N. J. Blair R. V. Alvarez X. (2019a). Comparative analysis of gammaherpesvirus circular RNA repertoires: conserved and unique viral circular RNAs. *J. Virol.* 93:e1952-18. 10.1128/JVI.01952-18 30567979 PMC6401440

[B155] Ungerleider N. A. Tibbetts S. A. Renne R. Flemington E. K. (2019b). Gammaherpesvirus RNAs Come Full Circle. *mBio* 10:e00071-19. 10.1128/mBio.00071-19 PMC6445933 30940699

[B156] Wan L. Zhang L. Fan K. Cheng Z. X. Sun Q. C. Wang J. J. (2016). Circular RNA-ITCH Suppresses Lung Cancer Proliferation via Inhibiting the Wnt/beta-Catenin Pathway. *Biomed. Res. Int.* 2016:1579490. 10.1155/2016/1579490 PMC5013215 27642589

[B157] Wang X. Zhang Y. Huang L. Zhang J. Pan F. Li B. (2015). Decreased expression of hsa_circ_001988 in colorectal cancer and its clinical significances. *Int. J. Clin. Exp. Pathol.* 8 16020–16025. 26884878 PMC4730091

[B158] Wang Y. Li H. Chan M. Y. Zhu F. X. Lukac D. M. Yuan Y. (2004). Kaposi’s sarcoma-associated herpesvirus ori-Lyt-dependent DNA replication: cis-acting requirements for replication and ori-Lyt-associated RNA transcription. *J. Virol.* 78 8615–8629. 10.1128/jvi.78.16.8615-8629.2004 15280471 PMC479094

[B159] Webster-Cyriaque J. Raab-Traub N. (1998). Transcription of Epstein-Barr virus latent cycle genes in oral hairy leukoplakia. *Virology* 248 53–65. 10.1006/viro.1998.9268 9705255

[B160] Weng W. Wei Q. Toden S. Yoshida K. Nagasaka T. Fujiwara T. (2017). Circular RNA ciRS-7-a promising prognostic biomarker and a potential therapeutic target in colorectal cancer. *Clin. Cancer Res.* 23 3918–3928. 10.1158/1078-0432.ccr-16-2541 28174233 PMC5511556

[B161] Wesselhoeft R. A. Kowalski P. S. Parker-Hale F. C. Huang Y. Bisaria N. Anderson D. G. (2019). RNA circularization diminishes immunogenicity and can extend translation duration in vivo. *Mol Cell* 74 508.e504–520.e504. 30902547 10.1016/j.molcel.2019.02.015 PMC6724735

[B162] White M. K. Pagano J. S. Khalili K. (2014). Viruses and human cancers: a long road of discovery of molecular paradigms. *Clin. Microbiol. Rev.* 27 463–481. 10.1128/cmr.00124-13 24982317 PMC4135891

[B163] Wu F. Cheng W. Zhao F. Tang M. Diao Y. Xu R. (2019). Association of N6-methyladenosine with viruses and related diseases. *Virol J.* 16:133. 10.1186/s12985-019-1236-3 PMC6849232 31711514

[B164] Wu J. Li Y. Wang C. Cui Y. Xu T. Wang C. (2019). CircAST: full-length assembly and quantification of alternatively spliced isoforms in circular RNAs. *Genomics Proteomics Bioinformatics* 17 522–534. 10.1016/j.gpb.2019.03.004 32007626 PMC7056934

[B165] Wu S. Liu S. Song H. Xia J. (2020). Circular RNA HIPK3 plays a carcinogenic role in cervical cancer progression via regulating miR-485-3p/FGF2 axis. *J. Investig. Med.* 69, 768–774. 10.1136/jim-2020-001537 33177072

[B166] Xia W. Qiu M. Chen R. Wang S. Leng X. Wang J. (2016). Circular RNA has_circ_0067934 is upregulated in esophageal squamous cell carcinoma and promoted proliferation. *Sci. Rep.* 6:35576. 10.1038/srep35576 PMC5067712 27752108

[B167] Xie H. Ren X. Xin S. Lan X. Lu G. Lin Y. (2016). Emerging roles of circRNA_001569 targeting miR-145 in the proliferation and invasion of colorectal cancer. *Oncotarget* 7 26680–26691. 10.18632/oncotarget.8589 27058418 PMC5042007

[B168] Xu L. Zhang M. Zheng X. Yi P. Lan C. Xu M. (2017). The circular RNA ciRS-7 (Cdr1as) acts as a risk factor of hepatic microvascular invasion in hepatocellular carcinoma. *J. Cancer Res. Clin. Oncol.* 143 17–27. 10.1007/s00432-016-2256-7 27614453 PMC11819007

[B169] Xuan L. Qu L. Zhou H. Wang P. Yu H. Wu T. (2016). Circular RNA: a novel biomarker for progressive laryngeal cancer. *Am. J. Transl. Res.* 8 932–939. 27158380 PMC4846937

[B170] Yamamoto T. Iwatsuki K. (2012). Diversity of Epstein-Barr virus BamHI-A rightward transcripts and their expression patterns in lytic and latent infections. *J. Med. Microbiol.* 61 1445–1453. 10.1099/jmm.0.044727-0 22700548

[B171] Yang J. Gong Y. Jiang Q. Liu L. Li S. Zhou Q. (2020). Circular RNA expression profiles in nasopharyngeal carcinoma by sequence analysis. *Front. Oncol.* 10:601. 10.3389/fonc.2020.00601 32426279 PMC7204547

[B172] Yang Y. Fan X. Mao M. Song X. Wu P. Zhang Y. (2017). Extensive translation of circular RNAs driven by N(6)-methyladenosine. *Cell Res.* 27 626–641. 10.1038/cr.2017.31 28281539 PMC5520850

[B173] Yao Z. Luo J. Hu K. Lin J. Huang H. Wang Q. (2017). ZKSCAN1 gene and its related circular RNA (circZKSCAN1) both inhibit hepatocellular carcinoma cell growth, migration, and invasion but through different signaling pathways. *Mol. Oncol.* 11 422–437. 10.1002/1878-0261.12045 28211215 PMC5527481

[B174] Yetming K. D. Lupey-Green L. N. Biryukov S. Hughes D. J. Marendy E. M. Miranda J. L. (2020). The BHLF1 locus of epstein-barr virus contributes to viral latency and B-cell immortalization. *J. Virol.* 94:e1215-20. 10.1128/JVI.01215-20 PMC7431786 32581094

[B175] Young L. S. Yap L. F. Murray P. G. (2016). Epstein-Barr virus: more than 50 years old and still providing surprises. *Nat. Rev. Cancer* 16 789–802. 10.1038/nrc.2016.92 27687982

[B176] Yu C. Y. Li T. C. Wu Y. Y. Yeh C. H. Chiang W. Chuang C. Y. (2017). The circular RNA circBIRC6 participates in the molecular circuitry controlling human pluripotency. *Nat. Commun.* 8:1149. 10.1038/s41467-017-01216-w PMC5658440 29074849

[B177] Yu L. Gong X. Sun L. Zhou Q. Lu B. Zhu L. (2016). The circular RNA Cdr1as act as an oncogene in hepatocellular carcinoma through targeting miR-7 expression. *PLoS One* 11:e0158347. 10.1371/journal.pone.0158347 27391479 PMC4938625

[B178] Yuan J. Cahir-Mcfarland E. Zhao B. Kieff E. (2006). Virus and cell RNAs expressed during Epstein-Barr virus replication. *J. Virol.* 80 2548–2565. 10.1128/jvi.80.5.2548-2565.2006 16474161 PMC1395376

[B179] Zapatka M. Borozan I. Brewer D. S. Iskar M. Grundhoff A. Alawi M. (2020). The landscape of viral associations in human cancers. *Nat. Genet.* 52 320–330. 10.1038/s41588-019-0558-9 32025001 PMC8076016

[B180] Zhang C. Zhang B. Yuan B. Chen C. Zhou Y. Zhang Y. (2020). RNA-Seq profiling of circular RNAs in human small cell lung cancer. *Epigenomics* 12 685–700. 10.2217/epi-2019-0382 32079426

[B181] Zhang X. O. Wang H. B. Zhang Y. Lu X. Chen L. L. Yang L. (2014). Complementary sequence-mediated exon circularization. *Cell* 159 134–147. 10.1016/j.cell.2014.09.001 25242744

[B182] Zhang Y. Zhang X. O. Chen T. Xiang J. F. Yin Q. F. Xing Y. H. (2013). Circular intronic long noncoding RNAs. *Mol. Cell.* 51 792–806. 10.1016/j.molcel.2013.08.017 24035497

[B183] Zhao J. Lee E. E. Kim J. Yang R. Chamseddin B. Ni C. (2019). Transforming activity of an oncoprotein-encoding circular RNA from human papillomavirus. *Nat. Commun.* 10:2300. 10.1038/s41467-019-10246-5 PMC6534539 31127091

[B184] Zhao Q. Chen S. Li T. Xiao B. Zhang X. (2018). Clinical values of circular RNA 0000181 in the screening of gastric cancer. *J. Clin. Lab. Anal.* 32:e22333. 10.1002/jcla.22333 28940688 PMC6817246

[B185] Zheng J. Liu X. Xue Y. Gong W. Ma J. Xi Z. (2017). TTBK2 circular RNA promotes glioma malignancy by regulating miR-217/HNF1beta/Derlin-1 pathway. *J. Hematol. Oncol.* 10:52. 10.1186/s13045-017-0422-2 PMC5319142 28219405

[B186] Zheng Y. Ji P. Chen S. Hou L. Zhao F. (2019). Reconstruction of full-length circular RNAs enables isoform-level quantification. *Genome Med.* 11:2. 10.1186/s13073-019-0614-1 PMC6339429 30660194

[B187] Zhu M. Liang Z. Pan J. Hu X. Zhang X. Xue R. (2020). HBV pgRNA can generate a circRNA with two junction sites. *bioRxiv [Preprint]* 10.1101/2020.05.14.095273

[B188] Zhu M. Xu Y. Chen Y. Yan F. (2017). Circular BANP, an upregulated circular RNA that modulates cell proliferation in colorectal cancer. *Biomed. Pharmacother.* 88 138–144. 10.1016/j.biopha.2016.12.097 28103507

